# Household energy poverty and trajectories of emotional and behavioural difficulties in children and adolescents: findings from two prospective cohort studies

**DOI:** 10.1007/s00127-024-02616-2

**Published:** 2024-02-12

**Authors:** David J. O Driscoll, Elizabeth Kiely, Linda M. O’Keeffe, Ali S. Khashan

**Affiliations:** 1 School of Public Health, University College Cork, Western Gateway Building, Cork, Ireland; 2 School of Applied Social Studies, William Thompson House, Donovan’s Road, Cork, Ireland; 3 MRC Integrative Epidemiology Unit, University of Bristol, Bristol, UK; 4 Population Health Sciences, Bristol Medical School, University of Bristol, Bristol, UK; 5 INFANT Research Centre, Cork University Hospital, University College Cork, Cork, Ireland

**Keywords:** Energy poverty, Poverty, Children, Adolescent, Psychopathology

## Abstract

**Purpose:**

The aim of this study is to examine the association between household energy poverty (EP) and trajectories of emotional and behavioural difficulties during childhood.

**Methods:**

The Growing up in Ireland study is two nationally representative prospective cohorts of children. The Infant Cohort (*n* = 11,134) were recruited at age 9 months (9 m) and followed up at 3, 5, 7 and 9 years (y). The Child Cohort (*n* = 8,538) were recruited at age 9 y and followed up at 13 y and 17/18 y. EP was a composite of two relative measures of EP. Emotional and behavioural difficulties were repeatedly measured using the strengths and difficulties questionnaire (SDQ). Linear spline multilevel models were used, adjusted for confounders to examine the association between (1) EP (9 m or 3 y) and trajectories of emotional and behavioural difficulties from 3 to 9 y in the Infant Cohort and (2) EP at 9 y and the same trajectories from 9 to 18 y in the Child Cohort.

**Results:**

In adjusted analyses, EP at 9 m or 3 y of age was associated with higher total difficulties score at 3 y (0.66, 95% CI 0.41, 0.91) and 5 y (0.77, 95% CI 0.48, 1.05) but not at 7 y or 9 y. EP at 9 y was associated with higher total difficulties score at 9 y (1.73, 95% CI 1.28, 2.18), with this difference reducing over time leading to 0.68 (95% CI 0.19, 1.17) at 17/18 y.

**Conclusions:**

Our study demonstrates a potential association between early life EP and emotional and behavioural difficulties that may be transient and attenuate over time during childhood. Further studies are required to replicate these findings and to better understand if these associations are causal.

**Supplementary Information:**

The online version contains supplementary material available at 10.1007/s00127-024-02616-2.

## Introduction

Energy inequality has received greater political attention internationally in recent years [10, 35, 40]. Housing is a social determinant of health and good housing incorporates thermal comfort, secure living conditions and access to local amenities [20]. Energy inequality (‘energy insecurity’, ‘energy poverty’, ‘fuel poverty’) has increased due to higher energy costs, rising inflation and the stock of poor energy efficient accommodation. Energy poverty may result in objective effects such as lower temperatures within homes, (i.e. condensation, dampness) and in subjective effects such as perceived inability to pay for heat.

There is growing evidence of the association between energy poverty and the development of physical and mental health difficulties as a child develops [25, 30, 37]. Despite this, energy poverty has been more extensively studied in older populations and there is a need for prospective cohorts examining energy poverty in children and adolescents and health outcomes (e.g. mental health, respiratory health, cognitive wellbeing) [5, 6]. Most studies investigating energy poverty focus on outcomes at a single time point and, there is limited understanding about how associations of energy poverty and outcomes change over time as a child develops (e.g. emotional and behavioural difficulties) [30]. This approach is needed to understand if the associations are transient or persistent as a child develops which in turn would provide supportive evidence to add to the growing literature that household energy poverty requires a public health and not just an economic solution.

In addition, a recent systematic scoping review by Champagne and colleagues highlighted the need for sex-specific analyses of the association of energy poverty with outcomes and they discussed that female adults may be at a higher risk of poor health outcomes following energy poverty exposure [6]. As such, there is a need to understand sex differences in outcomes following energy poverty exposure. Moreover, they highlight that energy poverty measures in the literature are inconsistent (e.g. housing quality, low-expenditure households, parental unemployment, cold homes, vulnerable groups) [6]. These raise an important point of ensuring that studies investigating energy poverty and an outcome are adequately adjusted for confounders for both the exposure and outcome in analysis (e.g. parental depression, home ownership, parental health, income) [19, 21, 39].

Using two nationally representative prospective cohorts, we examined the association between (1) energy poverty prior to 3 years of age and trajectories of emotional and behavioural difficulties from 3 to 9 years of age, (2) energy poverty at 9 years and the same trajectories from 9 to 18 years and (3) sex interaction analysis.

## Methods

### Data

Data were used from the Growing up in Ireland (GUI) study, which is comprised of a separate Infant and Child Cohort [33, 34]. Using the national child benefit register as a sampling frame, the Infant Cohort were recruited in 2008 at age 9 months (*n* = 11,134) and followed up at 3 (*n* = 9793), 5 (*n* = 9001), 7 (*n* = 5344) and 9 (*n* = 8032) years of age. The Infant Cohort is estimated to represent 1-in-9 children born in 2008 in Ireland (*n* = 11,194, included in wave 1). Using Irish national school records as a sampling frame, the Child Cohort was recruited in 2008 (born in 1998) at age 9 (*n* = 8538) years of age and followed up at ages 13 (*n* = 7495), and 17/8 (*n* = 6186) years of age. The Child Cohort is estimated to represent 1-in-7 children at 9 years of age.

The primary care giver (PCG) and children in later waves (9 years and older) were interviewed face-to-face using computer-aided personal interviewing by trained interviewers. Quail et al., provide a detailed description of the study design, interview method, and follow-up procedure [33, 34]. All waves had similar interview procedures except wave 4 (age 7) in the Infant Cohort which was a postal questionnaire. The authors received approval from the Central Statistics Office to use the RMF Child and Infant Cohort datasets. The GUI study received ethical approval from the Research Ethics Committee within the Irish Department of Health and Children, Ireland. All participants gave informed consent to enrol in the GUI study. This study is in accordance with the ethical standards as per the Declaration of Helsinki (1964) and subsequent amendments.

### Exposure

In the Infant Cohort, **‘energy poverty’** was classified as having energy poverty at 9 months or 3 years as we were interested in the effects of energy poverty in the sensitive period of early life, in particular prior to entering preschool or formal education. In the Child Cohort, the exposure **‘energy poverty’** was classified as having energy poverty at 9 years only. We used a previously reported approach of deriving relative energy poverty [24, 25]. Briefly, the primary caregiver was asked “*Does the household keep the home adequately warm?*” Either of the following responses “*no, cannot afford*” or “*no, other reason*” were constructed into a variable to represent “**cold home**”. The second question “*Have you ever had to go without heating during the last 12 months through lack of money?*” The response “*yes*” was used to construct into a variable to represent “**gone without heat**”. Therefore, the main exposure was **‘energy poverty’** which was a composite of either a “cold home” or “gone without heat”.

### Outcomes

#### Strengths and difficulties questionnaire (SDQ)

The SDQ was devised to identify current and early signs of emotional difficulties, hyperactivity, conduct behaviour, peer problems and prosocial issues in children [15]. Each question item has a three-point scale, ‘*not true*’, ‘*somewhat true*’ or ‘*certainly true*’ and each subscale has a total score (0–10). The use of SDQ as a continuous outcome has been validated in many jurisdictions [7, 13, 17, 22, 26]. The SDQ has strong psychometric properties and is used in many longitudinal cohorts [14, 36, 38]. We were interested in total difficulties, internalising, and externalising scores. The internalising score is the summed figure of emotional difficulties and peer problems. The externalising score is the summed score of hyperactivity and conduct behaviour scores. The total difficulties score was the total score of both the internalising and externalising scores. The internalising and externalising scores are appropriate in low-risk population samples. In the Infant Cohort, the PCG completed the SDQ for each wave, at 3, 5, 7 and 9 years of age. In the Child Cohort, the primary care giver completed the SDQ for each wave, at 9, 13 and 17/18 years of age. For repeated-measures analysis, we used continuous scores of SDQ total difficulties, internalising, and externalising scores.

### Confounders

A directed acyclic graph was used to identify the potential confounders (Fig. S1, Fig. S2). These included socioeconomic characteristics (household income, house ownership and household composition), primary care giver characteristics (age and educational status (obtained a degree), and primary care giver health-related characteristics measured before exposure (has a chronic health condition and depression symptom rating). All confounders were measured prior to exposure, including chronic health condition and depression symptom rating, which were measured prior to exposure in both cohorts.

### Statistical analysis

#### Primary analyses: repeated-measures analysis

We used linear spline multilevel modelling (2 levels: measurement occasion and individual) to examine the association between EP and trajectories of total, internalising and externalising SDQ scores in each cohort. Linear spline multilevel models estimate mean trajectories of the outcome while accounting for the non-independence (i.e. clustering) of repeated measurements within individuals and differences in the number and timing of measurements between individuals (using all available data from all eligible participants under a missing at random assumption) [27–29]. Linear splines allow knot points to be fit at different ages to derive periods in which change is approximately linear [28]. Linear spline periods were chosen to reflect ages in whole years that were closest to mean age at interview and hence where the density of measures was greatest. Age (in years) was centred at the first available measure (3 years for Infant Cohort and 9 years for Child Cohort). For the Child Cohort, we had two knots at 13 and 17/18 years; this produced two different linear slopes of the repeated outcome measure (i.e. SDQ): 9 to ≤ 13, and 13 to ≤ 17/18 years. For the Infant Cohort, we had three knots at 5, 7 and 9 years; this produced three different linear slopes of the repeated outcome measure (i.e. SDQ): 3 to ≤ 5, 5 to ≤ 7, and 7 to ≤ 9 years. All models included individual level random effects for the intercept and each linear spline period. For inclusion in the Infant Cohort analyses, participants required data on EP prior to 3 years with at least one measure of SDQ from 3 to 9 years and complete confounder data. For inclusion in the Child Cohort analyses, participants required data on EP at 9 years with at least one measure of SDQ from 9 to 17/18 years and complete confounder data. The growth parameters (SDQ) subsequently vary following exposure to EP. Model fit statistics compared observed values of each outcome at each age with those predicted by the models. The difference in mean or rate of change was calculated by subtracting estimates between EP and no EP (STATA syntax is available in Supplementary material 2). All data analysis was carried out using the statistical software package STATA (v.17).

#### Sensitivity analyses

In the Infant Cohort, we examined whether associations of EP at 9 months only, 3 years only, and 9 months and 3 years combined were similar to the results of our main analysis (EP at 9 months or 3 years). In both cohorts, we also examined whether associations of ‘cold home’ or ‘gone without heat’ separately were similar to results of our main analysis (which was a composite of these). We compared participants include to those not included in the analysis for each cohort. We examined whether our results differed by gender by performing an interaction analysis using both cohorts. We provide descriptive analysis of the outcome variable over time at each age and number of outcome measurements per participant included in analysis. Finally, we repeated the analysis examining the associations of EP between 3 and 9 years in the Infant Cohort and 9 and 17/18 years in the Child Cohort with a measure of complete outcomes in each Cohort.

## Results

### Summary of demographics of Infant Cohort

Of 10 170 included in the Infant Cohort analysis, 16.9% (*n* = 1 473) was exposed to energy poverty (Table 1). Participants that experienced EP prior to 3 years of age had higher levels of single parent households, depression scores, lower levels of household income and home ownership and educational attainment compared with participants that did not experience EP prior to 3 years of age. Gender was similar between the groups.

**Table 1:** Summary of demographic covariates and outcomes of participants used for analysis in the Irish Growing Up in Ireland—Infant Cohort (*n* = 10,170)

	No energy poverty		Energy poverty	
	(*n* = 8697)		(*n* = 1473)	
	*n*	(%)	*n*	(%)
**Covariates**				
Gender (female)	4 279	(49.2)	725	(49.2)
4 Category household type				
1 parent, 1 child	380	(4.4)	127	(8.6)
1 parent, 2 + child	429	(4.9)	225	(15.3)
2 parents, 1 child	2 990	(34.4)	344	(23.4)
2 parents, 2 + child	4 898	(56.3)	777	(52.7)
Equivalised household income (quintile)				
1st	1 323	(15.2)	599	(40.7)
2nd	1 389	(16)	354	(24)
3rd	1 624	(18.7)	208	(14.1)
4th	1 944	(22.4)	131	(8.9)
5th	1 764	(20.3)	83	(5.6)
Home owner	6 398	(73.6)	616	(41.8)
Relationship of PCG to child (parent)	8 697	(100)	1 472	(99.9)
PCG gender (female)	8 672	(99.7)	1 467	(99.6)
PCG age (yrs)				
< 26	1272	(14.6)	438	(29.7)
27–30	1690	(19.4)	362	(24.6)
31–35	3290	(37.8)	389	(26.4)
36–39	1838	(21.1)	204	(13.8)
40 +	607	(7)	80	(5.4)
PCG degree	3443	(39.6)	299	(20.3)
PCG chronic health problem	939	(10.8)	227	(15.4)
PCG depression	751	(8.6)	289	(19.6)
**Outcome (mean (SD))**				
Total SDQ at 3 yrs	7.6	(4.4)	9.2	(5.0)
Total SDQ at 5 yrs	7.0	(4.6)	8.6	(5.3)
Total SDQ at 7 yrs	7.0	(5.2)	8.6	(5.9)
Total SDQ at 9 yrs	6.9	(5.2)	8.8	(6.1)

*SDQ* strengths and difficulties questionnaire, *PCG* primary care giver, *yrs* years, *m* months, *SD* standard deviation

### Summary of demographics of Child Cohort

Of 8 560 included in the Child Cohort analysis, 5.3% (*n* = 451) was exposed to energy poverty (Table 2). Participants who experienced EP at 9 years of age had higher levels of single parent households, depression scores, lower levels of household income, and home ownership, and educational attainment compared with participants who did not experience EP at 9 years of age. Gender was similar between the groups.

**Table 2:** Summary of demographic covariates and outcomes of participants used for analysis in the Irish Growing Up in Ireland—Child Cohort (*n* = 8560)

	No energy poverty		Energy poverty	
	(*n* = 8109)		(*n* = 451)	
	*n*	(%)	*n*	(%)
**Covariates**				
Gender (female)	4151	(51.2)	249	(55.2)
4 Category household type				
1 parent, 1 child	605	(7.5)	71	(15.7)
1 parent, 2 + child	270	(3.3)	44	(9.8)
2 parents, 1 child	3162	(39)	130	(28.8)
2 parents, 2 + child	4072	(50.2)	206	(45.7)
Equivalised household income (quintile)				
1st	910	(11.2)	143	(31.7)
2nd	1288	(15.9)	87	(19.3)
3rd	1515	(18.7)	66	(14.6)
4th	1761	(21.7)	53	(11.8)
5th	2036	(25.1)	73	(16.9)
Home owner	6853	(84.5)	292	(64.7)
Relationship of PCG to child (parent)	8084	(99.7)	448	(99.3)
PCG gender (female)	8014	(98.8)	444	(98.4)
PCG age (yrs.)				
< 30^a^	483	(5.9)	48	(10.7)
31–35	1 098	(13.5)	82	(18.2)
36–39	1 989	(24.5)	115	(25.5)
40 +	4 539	(56)	206	(45.7)
PCG degree	2 147	(26.5)	89	(19.7)
PCG chronic health problem	1 041	(12.8)	83	(18.4)
PCG depression	565	(7)	63	(14)
**Outcome (mean (SD))**				
Total SDQ at 9 yrs	7.3	(4.9)	9.8	(6.2)
Total SDQ at 13 yrs	6.4	(5.0)	7.8	(5.7)
Total SDQ at 17/18 yrs	6.4	(4.9)	7.7	(5.7)

*SDQ* strengths and difficulties questionnaire, *PCG* primary care giver, *yrs* years, *m* months, *SD* standard deviation

^a^Less than 26 years was combined with 27–30 years to comply with statistical non-disclosure procedures

### Primary analyses

**Fig. 1:**
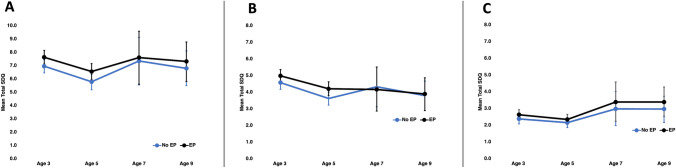
Trajectories of (**a**) total difficulties score, (**b**) externalising difficulties score, and (**c**) internalising difficulties score from 3 to 9 years by energy poverty (EP) exposure prior to 3 years, from adjusted analysis (primary care giver age, primary care giver education, household composition, household income, home ownership, primary care giver chronic health status and depression status) using the Irish Growing Up in Ireland—Infant Cohort

Mean trajectories of total difficulties, internalising and externalising scores from 3 to 9 years by EP prior to age 3 are shown in Fig. 1. In fully adjusted models in the Infant Cohort, EP prior to 3 years was associated with a higher total difficulties scores at 3 years (0.66, 95% CI 0.41, 0.91), 5 years (0.77, 95% CI 0.48, 1.05) but not 7 years (0.26, 95% CI −0.77, 1.29) or 9 years (0.52, 95% CI −0.23, 1.27) of age (Table 3). Energy poverty prior to 3 years of age was associated with higher internalising scores at 3 years (0.24, 95% CI 0.12, 0.37) and 5 years (0.19, 95% CI 0.04, 0.33) and the results spanned the null value at 7 years (0.40, 95% CI −0.20, 1.00) and 9 years (0.41, 95% CI −0.02, 0.85) of age. Similarly, EP prior to 3 years of age was associated with higher externalising scores at 3 years (0.41, 95% CI 0.23, 0.59) and 5 years (0.58, 95% CI 0.38, 0.78) but not 7 years (−0.15, 95% CI −0.85, 0.55) or 9 years (0.09, 95% CI −0.40, 0.60) of age.

**Table 3:** Mean trajectories of total difficulties, externalising and internalising scores at 3, 5, 7 and 9 years of age by energy poverty prior to 3 years of age using the Irish Growing Up in Ireland—Infant Cohort

	Unadjusted			Adjusted		
	No EP	EP	No EP vs. EP	No EP	EP	No EP vs. EP
SDQ	Mean trajectory (95% CI)	Mean trajectory (95% CI)	Mean difference in trajectory comparing EP to no EP (95% CI)	Mean trajectory (95% CI)	Mean trajectory (95% CI)	Mean difference in trajectory comparing EP to no EP (95% CI
* Total SDQ difficulties score*						
MD Age 3 yrs	7.57 (7.48, 7.67)	9.17 (8.94, 9.39)	1.59 (1.34, 1.84)	6.96 (6.47, 7.45)	7.63 (7.1, 8.15)	0.66 (0.41,0.91)
$\Delta$ 3 to 5 yrs	−0.28(−0.33, 0.23)	−0.24 (−0.37,−0.13)	0.03 (−0.10, 0.16)	−0.58 (−0.85, −0.32)	−0.53 (−0.81, −0.24)	0.05 (−0.08, 0.19)
MD Age 5 yrs	7.01 (6.90, 7.12)	8.67 (8.41, 8.93)	1.66 (1.38, 1.94)	5.79 (5.24, 6.34)	6.56 (5.96, 7.16)	0.77 (0.48, 1.05)
$\Delta$ 5 to 7 yrs	0.42 (0.26, 0.58)	0.59 (0.11, 1.08)	0.17 (−0.34, −0.68)	0.77 (−0.12, 1.68)	0.52 (−0.49, 1.54)	−0.25 (−0.78, 0.27)
MD Age 7 yrs	7.86 (7.54, 8.18)	9.86 (8.91, 10.81)	2.00 (1.00, 3.00)	7.35 (5.58, 9.12)	7.61 (5.63, 9.59)	0.26 (−0.77, 1.29)
$\Delta$ 7 to 9 yrs	−0.14 (−0.19, −0.9)	−0.12 (−0.28, 0.05)	0.03 (−0.15, 0.20)	−0.27 (−0.59, 0.03)	−0.14 (−0.49, 0.20)	0.12 (−0.05, 0.31)
MD Age 9 yrs	7.57 (7.34, 7.81)	9.63 (8.94, 10.32)	2.05 (1.32, 2.79)	6.80 (5.48, 8.11)	7.32 (5.85, 8.78)	0.52 (−0.23, 1.27)
*SDQ externalising score*						
MD Age 3 yrs	5.15 (5.08, 5.21)	6.17 (6.00, 6.32)	1.01 (0.84, 1.19)	4.58 (4.23, 4.93)	4.99 (4.62, 5.37)	0.41 (0.23, 0.59)
$\Delta$ 3 to 5 yrs	−0.27 (−0.31, −0.24)	−0.22 (−0.31,−0.13)	0.05 (−0.03, 0.14)	−0.47 (−0.66, −0.28)	−0.38 (−0.59, −0.18)	0.08 (−0.00, 0.18)
MD Age 5 yrs	4.59 (4.52, 4.66)	5.71 (5.53, 5.90)	1.12 (0.92, 1.32)	3.63 (3.24, 4.01)	4.21 (3.79, 4.63)	0.58 (0.38, 0.78)
$\Delta$ 5 to 7 yrs	−0.12 (−0.23, −0.01)	−0.32 (−0.65, 0.00)	−0.19 (−0.54, 0.14)	0.35 (−0.26, 0.96)	−0.02 (−0.71, 0.67)	−0.37 (−0.73, −0.00)
MD Age 7 yrs	4.33 (4.12, 4.55)	5.06 (4.42, 5.71)	0.72 (0.05, 1.40)	4.33 (3.13, 5.52)	4.17 (2.83, 5.52)	−0.15 (−0.85, 0.55)
$\Delta$ 7 to 9 yrs	−0.12 (−0.16, −0.08)	−0.05 (−0.16, 0.06)	0.07 (−0.05, 0.19)	−0.26 (−0.48, −0.04)	−0.13 (−0.38, 0.10)	0.12 (−0.00, 0.25)
MD Age 9 yrs	4.09 (3.93, 4.25)	4.96 (4.50, 5.42)	0.87 (0.38, 1.35)	3.80 (2.94, 4.67)	3.90 (2.94, 4.87)	0.09 (−0.40, 0.60)
* SDQ internalising score*						
MD Age 3 yrs	2.42 (2.37, 2.47)	2.99 (2.88, 3.11)	0.57 (0.45, 0.69)	2.38 (2.13, 2.63)	2.63 (2.36, 2.90)	0.24 (0.12, 0.37)
$\Delta$ 3 to 5 yrs	−0.00 (−0.03, 0.02)	−0.02 (−0.09, 0.04)	−0.01 (−0.09, 0.05)	−0.11 (−0.26, 0.04)	−0.14 (−0.30, 0.02)	−0.02 (−0.10, 0.04)
MD Age 5 yrs	2.41 (2.36, 2.46)	2.95 (2.82, 3.08)	0.53 (0.39, 0.68)	2.16 (1.87, 2.44)	2.35 (2.04, 2.65)	0.19 (0.04, 0.33)
$\Delta$ 5 to 7 yrs	0.53 (0.44, 0.63)	0.90 (0.61, 1.18)	0.36 (0.06, 0.06)	0.41 (−0.12, 0.95)	0.51 (−0.07, 1.11)	0.10 (−0.20, 0.41)
MD Age 7 yrs	3.49 (3.30, 3.81)	4.75 (4.20, 5.31)	1.26 (0.68, 1.85)	2.98 (1.95, 4.02)	3.39 (2.23, 4.54)	0.40 (−0.20, 1.00)
$\Delta$ 7 to 9 yrs	−0.01 (−0.04, 0.01)	−0.05 (−0.15, 0.03)	−0.04 (−0.14, 0.05)	−0.00 (−0.19, 0.17)	0.00 (−0.20, 0.20)	0.00 (−0.09, 0.11)
MD Age 9 yrs	3.45 (3.32, 3.59)	4.63 (4.23, 5.04)	1.18, (0.75, 1.60)	2.97 (2.21, 3.73)	3.39 (2.54, 4.24)	0.41 (−0.02, 0.85)

**Adjusted for:** household income, household composition, household home owner, primary care giver (PCG) age, PCG education, PCG chronic illness and PCG depression. MD mean difference in SDQ score $\Delta$ mean difference in change per year of SDQ score

*yrs* years of age, *SDQ* strengths and difficulties questionnaire, *EP* energy poverty, *CI* 95% confidence interval

**Fig. 2:**
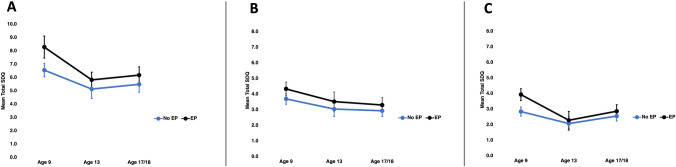
Trajectories of (**a**) total difficulties score, (**b**) externalising difficulties score, and (**c**) internalising difficulties score from 9 years to 17/18 years by energy poverty (EP) exposure at 9 years, from adjusted analysis (primary care giver age, primary care giver education, household composition, household income, home ownership, primary care giver chronic health status and depression status) using the Irish Growing Up in Ireland—Child Cohort

Mean trajectories of total difficulties, internalising and externalising scores from 9 years to 17/18 years by EP at 9 years are shown in Fig. 2. In fully adjusted models in the Child Cohort, EP at 9 years of age was associated with a higher total difficulties scores at 9 years (1.73, 95% CI 1.28, 2.18) with evidence of weakening associations at 13 years (0.69, 95% CI 0.05, 1.33) and also subsequently 17/18 years (0.68, 95% CI 0.19, 1.17) of age. Energy poverty at 9 years of age was associated with a higher externalising scores at 9 years (0.63, 95% CI 0.34, 0.93) and internalising scores at 9 years (1.09, 95% CI 0.83, 1.35). These patterns of association were weaker than associations for total difficulties scores but similarly indicated evidence of weakening or attenuating by 18 years of age (Table 4).

**Table 4:** Mean trajectories of total difficulties, externalising and internalising scores at 9, 13 and 17/18 years of age by energy poverty at 9 years of age using the Irish Growing Up in Ireland—Child Cohort

	Unadjusted			Adjusted		
	No EP	EP	No EP vs. EP	No EP	EP	No EP vs. EP
SDQ	Mean trajectory (95% CI)	Mean trajectory (95% CI)	Mean difference in trajectory comparing EP to no EP (95% CI)	Mean trajectory (95% CI)	Mean trajectory (95% CI)	Mean difference in trajectory comparing EP to no EP (95% CI)
* Total SDQ difficulties score*						
MD Age 9 yrs	7.25 (7.15, 7.36)	9.81 (9.36, 10.27)	2.55 (2.09, 3.02)	6.56 (6.05, 7.07)	8.29 (7.63, 8.96)	1.73 (1.28, 2.18)
$\Delta$ 9 to 13 yrs	−0.41 (−0.48, −0.34)	−0.92 (−1.22, −0.62)	−0.51 (−0.81, −0.20)	−0.71 (−1.05, −0.36)	−1.23 (−1.68, −0.77)	−0.52 (−0.82, −0.21)
MD Age 13 yrs	6.43 (6.28, 6.57)	7.96 (7.34, 8.59)	1.53 (0.89, 2.18)	5.14 (4.41, 5.86)	5.83 (4.89, 6.77)	0.69 (0.05, 1.33)
$\Delta$ 13 to 17/18 yrs	0.01 (−0.01, 0.04)	−0.01 (−0.14, 0.11)	−0.03 (−0.16, −0.09)	0.18 (0.03, 0.33)	0.18 (−0.01, 0.37)	−0.00 (−0.13, 0.12)
MD Age 17/18 yrs	6.46 (6.35, 6.57)	7.94 (7.45, 8.43)	1.47 (0.97, 1.97)	5.50 (4.95, 6.06)	6.19 (5.46, 6.92)	0.68 (0.19, 1.17)
*SDQ externalising score*						
MD Age 9 yrs	4.18 (4.11, 4.25)	5.21 (4.91, 5.50)	1.02 (0.72, 1.32)	3.70 (3.37, 4.04)	4.34 (3.91, 4.77)	0.63 (0.34, 0.93)
$\Delta$ 9 to 13 yrs	−0.18 (−0.22, −013)	−0.22 (−0.41, −0.03)	−0.04 (−0.23, 0.14)	−0.32 (−0.54, −0.10)	−0.40 (−0.69, −0.11)	−0.07 (−0.27, 0.11)
MD Age 13 yrs	3.82 (3.73, 3.91)	4.75 (4.35, 5.16)	0.93 (0.52, 1.34)	3.05 (2.59, 3.52)	3.53 (2.92, 4.14)	0.47 (0.06, 0.89)
$\Delta$ 13 to 17/18 yrs	−0.09 (−0.11, −0.07)	−0.17 (−0.25, −0.09)	−0.07 (−0.16, 0.00)	−0.05 (−0.14, 0.03)	−0.11 (−0.23, 0.00)	−0.05 (−0.13, 0.02)
MD Age 17/18 yrs	3.62 (3.55, 3.70)	4.40 (4.09, 4.71)	0.77 (0.45, 1.09)	2.94 (2.58, 3.30)	3.31 (2.84, 3.78)	0.36 (0.04, 0.68)
* SDQ internalising score*						
MD Age 9 yrs	3.07 (3.01, 3.13)	4.60 (4.34, 4.86)	1.52 (1.26, 1.79)	2.85 (2.56, 3.14)	3.94 (3.56, 4.32)	1.09 (0.83, 1.35)
$\Delta$ 9 to 13 yrs	−0.23 (−0.27, −0.18)	−0.69 (−0.88, −0.51)	−0.46 (−0.65, −0.27)	−0.38 (−0.60, −0.16)	−0.82 (−1.11, −0.53)	−0.44 (−0.63, −0.24)
MD Age 13 yrs	2.61 (2.52, 2.69)	3.20 (2.83, 3.58)	0.59 (0.20, 0.98)	2.08 (1.65, 2.52)	2.29 (1.72, 2.86)	0.20 (−0.17, 0.59)
$\Delta$ 13 to 17/18 yrs	0.11 (0.09, 0.13)	0.16 (0.07, 0.24)	0.04 (−0.03, 0.13)	0.23 (0.14, 0.33)	0.28 (0.16, 0.41)	0.05 (−0.03, 0.13)
MD Age 17/18 yrs	2.83 (2.77, 2.90)	3.52 (3.24, 3.80)	0.68 (0.40, 0.97)	2.56 (2.24, 2.88)	2.87 (2.45, 3.29)	0.31 (0.02, 0.59)

**Adjusted for:** household income, household composition, household home owner, primary care giver (PCG) age, PCG education, PCG chronic illness and PCG depression

MD mean difference in SDQ score ∆ mean difference in change per year of SDQ score

*yrs* years of age, *SDQ* strengths and difficulties questionnaire, *EP* energy poverty, *CI* 95% confidence interval

### Sensitivity analyses

Associations of EP at 9 months only and trajectories were similar to main analysis. Energy poverty at 3 years only was associated with a higher total difficulties score at each age across the trajectory from 3 to 9 years of age compared with our main analysis (Table S1). The analysis of the variables ‘*cold home*’ and ‘*gone without heat*’ are available in Table S2, Table S3, and Table S4. This analysis demonstrated that each on its own did not explain the ‘energy poverty’ composite variable result. Participants included in our Infant Cohort analysis (*n* = 10,170) had a higher proportion of females, higher proportions of 2 parent families, higher household income and home ownership compared with participants excluded from our analyses (*n* = 964) (Table S5). In the Child Cohort, only (*n* = 8) were not included in analysed sample and we, therefore, did not have sufficient excluded numbers to examine characteristics of included versus excluded participants.

Energy poverty prior to 3 years was associated with higher total difficulties scores at 3 years (difference: 1.45, 95% CI 1.14, 1.75) and 5 years (difference: 0.66, 95% CI 0.41, 0.91) in girls compared with boys ((Table S6, Fig. S3) but these sex differences attenuated thereafter at age 7 and 9. Energy poverty at 9 years in the Child Cohort resulted in higher total difficulties scores at 9 years in girls (difference: 2.44, 95% CI 1.94, 2.93) compared to boys (difference: 1.73, 95% CI 1.29, 2.19). Contrastingly, at age 13 years, there was a higher total difficulties scores in boys (difference: 1.12, 95% CI 0.29, 1.94) compared to girls (difference: 0.60, 95% CI -0.30, 1.50), and again at age 17/18 years in boys (difference: 1.00, 95% CI 0.38, 1.62) compared to girls (difference: 0.24, 95% CI −0.43, 0.91) (Table S7, Fig. S4). Table S8 and Table S9 describe the outcome variable over time at each age and the number of outcome measurements per participant. The mean (standard deviation) number of available SDQ outcomes per participant was 2.9 (1.3) in the Infant Cohort and 2.6 (0.7) in the Child Cohort. Complete case analysis of SDQ outcome showed similar results to our main analysis for both the Child and Infant Cohorts (Table S10 and Table S11).

## Discussion

In this large Irish prospective cohort study, we found that children, if exposed to energy poverty prior to 3 years of age may have a transient higher caregiver reported internalising and externalising scores at age 3, and 5 but not 7 or 9 years of age. Contrastingly, energy poverty at 9 years of age was associated with higher internalising and externalising scores at age 9, 13, and 17/18 years of age. Total difficulties scores showed similar results, energy poverty prior to 3 years was associated with higher total difficulties scores at 3 and 5 years but not at 7 or 9 years of age. Energy poverty at 9 years was associated with higher total difficulties scores at 9 years with this difference reducing over time at 17/18 years.

First, we have demonstrated an association between energy poverty exposure and childhood emotional and behavioural difficulties. This is similar to Fernandez et al., who demonstrated a higher odds of internalising and externalising behaviours in 9 year olds exposed to dual food and energy poverty in the previous 12-month period [9]. Clinically, the awareness of poverty exposures (e.g. energy poverty) is important to appreciate reported childhood emotional and behavioural difficulties.

Second, our analyses provides preliminary evidence that energy poverty exposure may result in a marginally higher total difficulties score suggesting worse emotional and behaviour difficulties. Positively, the total difficulties score does not worsen over time, nor does the rate of change in total difficulties score in those children exposed to energy poverty prior to 3 years of age. It is possible to postulate, older children exposed to energy poverty may have had a longer exposure period and be more susceptible to perceived poverty. This may explain the higher total difficulties scores at 9, 13 and 17/18 years in those exposed to energy poverty at 9 years of age. To the best of our knowledge, this is the first study to investigate energy poverty exposure and trajectory of emotional and behavioural wellbeing longitudinally during early and late childhood.

Thirdly, the mechanism that explains our findings is not well understood and it could be the case that families may prioritise a ‘warm home’ if they have children despite the cost [2]. It is possible that children in energy poverty exposure may have a poorer quality of home, and as such spend a greater amount of time outdoors. According to Gold [12], poor housing may lead to higher externalising and conduct-related issues [12]. Furthermore, the mixed method “Cool? Study” in New Zealand, demonstrated that almost half the adolescents studied felt their home was not warm enough during the winter months, and that adolescents restrict their activities due to a cold home, e.g. invite friends over to their house, or do their homework [32]. Moreover, externalising behaviours are associated with poor academic performance [1, 4, 11, 23]. There is emerging evidence of gender behavioural differences in young people exposed to different poverty exposures [18]. According to our findings, we cannot conclude that the associations were sex specific. Albeit, females in comparison to males had higher total and externalising difficulties initially following exposure. However, these results warrant further studies to understand if males or females moderate the level of externalising behaviours (e.g. school refusal, truancy, conduct-related behaviours) due to energy poverty or some other unknown aspect of poverty.

This study contains limitations. First, the exposure variable ‘energy poverty’ was subjectively measured and is also a composite variable. The exposure variable is also subject to recall bias by the primary care giver, although, a similar approach has been used in other studies [31, 37] and relative or subjective measures of energy poverty are used in the European Union’s Survey of Income and Living Conditions (EU-SILC) [8, 37]. Relative binary energy poverty measures do not provide exact temperature or cost data, albeit it provides the family centred feedback on the sense of being in energy poverty [3]. Moreover, this interpretation provides information of the lived and perceived household experience of relative energy poverty. Second, the SDQ outcomes are dependent on the subjective evaluation by a caregiver. However, these questionnaires have good correlation with identifying children requiring further investigation [26, 38]. We did not use the prosocial scale of the SDQ as it has a poor correlation with the other subscales and as such, was not incorporated in the analysis. Third, families with mental health and behavioural issues may be more likely to be lost to follow-up in longitudinal studies. This may also be due to poor educational attainment, challenges pertaining to ethnic status and employment mobility. This is particularly important when attempting to capture components of poverty. Fourth, we attempted to control for all known and available confounders following the literature review. However, residual confounding may persist due to unmeasured confounding, Potential unmeasured confounders may include household heating type, accommodation size and accommodation quality (including energy efficiency rating). Fifth, energy poverty may be a component of underlying overall household poverty that influences differences in SDQ and may reflect overall socioeconomic household circumstances. Finally, the available dataset utilised is not dated in the context of recent increases in energy costs, rising inflation and ongoing coronavirus pandemic implications. Indeed, the current global energy crisis seems likely to further exacerbate existing energy inequalities internationally.

The strengths of this study includes using a large contemporary and relatively recent nationally representative cohort, the use of repeated-measures analysis to maximise potential participants, reduce loss to follow-up and selection bias. Moreover, the study outcomes were measured prospectively.

While further research is required to better understand the association between energy poverty and childhood emotional and behavioural outcomes, this study highlights the need for wider societal and public policy to understand and potentially alleviate material deprivation (including energy poverty). As Mohan [25] argued, a blended approach to poverty reform is needed, that incorporates direct short-term mechanisms of relief and long-term strategies to lift each subsequent generation from experienced and lived poverty [25]. A short-term mechanism may include energy vouchers and long-term strategies may include upgrading housing (private and social) to energy efficient standards with renewable resources to lessen the long-term risk of a household remaining in poverty or returning to poverty. Furthermore, a strategic focus on policies that target energy poverty households specifically is required and this may also require longitudinal tracking of energy poverty households and the health conditions of each household member over time to inform policy decision making and efficacy.

Health clinicians and educators should be aware of the present risk of externalising and internalising difficulties if a child is actively exposed to energy poverty. This is a collective reminder of the importance of addressing active social deprivation and taking a careful approach to addressing behavioural difficulties and inherent poverty at the same time. This research demonstrates that early life exposure to energy related deprivation has an association with difficulties in children. Although, we identified energy poverty exposure with emotional and behavioural outcomes in children, energy poverty exposure may be a proxy measure of wider socioeconomic factors within a child’s life. As such, government intervention in addressing material deprivation (including energy poverty) for young children may have later life protective effects in these children’s emotional and behavioural development, and potentially reduce future care or intervention costs.

From a research perspective, more objective and subjective metrics of energy poverty as an exposure are needed. There is a need to understand the longitudinal effect of constant and frequent transitioning into energy poverty on a child’s emotional and behavioural development and if it is a mediator or moderator of academic performance and attainment. Finally, energy poverty occurs within households and within neighbourhoods as such a multilevel approach to inform holistic poverty intervention polices (i.e. integrated public health, housing and welfare interventions) is greatly needed [16].

## Conclusion

In this large prospective nationally representative cohort, we demonstrated that energy poverty exposure is associated with emotional and behavioural difficulties at several ages across early and late childhood. Further work is required to better understand these associations, specifically whether these associations are causal or whether energy poverty exposure association is being driven by wider socioeconomic deprivation. Such studies would have implications for further policy development and interventions to address energy poverty, which is an increasing problem internationally.

### Supplementary Information

Below is the link to the electronic supplementary material.

Supplementary file1 (DOCX 4631 KB)

Supplementary file2 (DOCX 19 KB)

## Data Availability

The data analysed in this study may be accessed by applying for either the Anonymised Microdata File (AMF) and/or the Researcher Microdata File (RMF) through the Irish Social Science Data Archive and/or Central Statistics Office respectively.
