# Supplementary material for: Household energy poverty and trajectories of emotional and behavioural difficulties in children and adolescents: findings from two prospective cohort studies
Source: Soc Psychiatry Psychiatr Epidemiol. 2024 Feb 12;59(8):1299–309. doi: 10.1007/s00127-024-02616-2 (PMC11291537; doi:10.1007/s00127-024-02616-2)

**Supplementary material**

**Title:** Household energy poverty and trajectories of emotional and behavioural difficulties in children and adolescents: findings from two prospective cohort studies

**David J O Driscoll^1^, Elizabeth Kiely^2^, Linda M O’ Keeffe^1,4,5^, Ali S Khashan^1,3^.**

**Table S1:** Mean trajectories of total difficulties, externalising and internalising scores at 3, 5, 7 and 9 years of age by energy poverty exposure at combined 9 months and 3 years, 9 months only, and 3 years only using the Irish Growing Up in Ireland – Infant Cohort.

**Table S2:** Mean trajectories of total difficulties scores at 3, 5, 7 and 9 years of age by ‘Gone without Heat’ exposure at 9 months only, 3 years only and combined 9 months or 3 years of age using the Irish Growing Up in Ireland – Infant Cohort.

**Table S3:** Mean trajectories of total difficulties scores at 3, 5, 7 and 9 years of age by ‘Cold Home’ exposure at 9 months only, 3 years only and combined 9 months or 3 years of age using the Irish Growing Up in Ireland – Infant Cohort.

**Table S4:** Mean trajectories of total difficulties scores at 9, 13 and 17/18 years of age by ’Gone without Heat’ and ‘Cold Home’ exposure at 9 years of age using the Irish Growing Up in Ireland – Child Cohort.

**Table S5:** Comparison of household and caregiver characteristics included in analysis (n=10 170) and excluded from analysis (n=964) using the Irish Growing Up in Ireland – Infant Cohort.

**Table S6**: Mean trajectories, sex stratification and interaction effects of total difficulties, externalising and internalising scores at 3, 5, 7 and 9 years of age by energy poverty prior to 3 years of age using the Irish Growing Up in Ireland – Infant Cohort.

**Table S7:** Mean trajectories, sex stratification and interaction effects of total difficulties, externalising and internalising scores at 9, 13 and 17/18 years of age by energy poverty at 9 years of age using the Irish Growing Up in Ireland – Child Cohort.

**Table S8:** Description of outcome (strengths and difficulties questionnaire) included at each age and the number of outcomes used in analysis per participant using the Irish Growing Up in Ireland – Infant Cohort (n=11 134).

**Table S9:** Description of outcome (strengths and difficulties questionnaire) included at each age and the number of outcomes used in analysis per participant using the Irish Growing Up in Ireland – Child Cohort (n=8 568).

**Table S10:** Mean trajectories of total difficulties scores from 3 to 9 years of age by energy poverty prior to 3 years of age using the Irish Growing Up in Ireland – Infant Cohort (n=4 730), including participants with an outcome measure at each wave.

**Table S11:** Mean trajectories of total difficulties scores from 9 to 17/18 years of age by energy poverty prior to 9 years of age using the Irish Growing Up in Ireland – Child Cohort (n= 5 958), including participants with an outcome measure at each wave.

**Figure S1**: Directed acyclic graph (DAG) of study design using Irish Growing Up in Ireland Infant Cohort.

**Figure S2**: Directed acyclic graph (DAG) of study design using Irish Growing Up in Ireland Child Cohort.

**Figure S3:** Trajectories of (A) total difficulties score, (B) externalising difficulties score, and (C) internalising difficulties score from 3 years to 9 years by energy poverty exposure in males (M-EP) and females (F-EP) prior to 3 years of age, from adjusted analysis (primary care giver age, primary care giver education, household composition, household income, home ownership, primary care giver chronic health status and depression status using the Growing Up in Ireland – Infant Cohort.

**Figure S4:** Trajectories of (A) total difficulties score, (B) externalising difficulties score, and (C) internalising difficulties score from 9 years to 17/18 years by energy poverty exposure in males (M-EP) and females (F-EP) at 9 years, from adjusted analysis (primary care giver age, primary care giver education, household composition, household income, home ownership, primary care giver chronic health status and depression status using the Growing Up in Ireland – Child Cohort.

**Tables**

**Table S1:** Mean trajectories of total difficulties, externalising and internalising scores at 3, 5, 7 and 9 years of age by energy poverty exposure at combined 9 months and 3 years, 9 months only, and 3 years only using the Irish Growing Up in Ireland – Infant Cohort.

|  | **Unadjusted** | | | **Adjusted** | | |
| --- | --- | --- | --- | --- | --- | --- |
|  | **No EP** | **EP** | **No EP vs EP** | **No EP** | **EP** | **No EP vs EP** |
| **tSDQ** | **Mean trajectory (95% CI)** | **Mean trajectory (95% CI)** | **Mean difference in trajectory (95% CI)** | **Mean trajectory (95% CI)** | **Mean trajectory (95% CI)** | **Mean difference in trajectory comparing EP to no EP**  **(95% CI)** |
|  | **Energy Poverty at 9 months and 3 years** | | | | | |
| MD Age 3 yrs. | 7.68 (7.59, 7.77) | 10.28(9.75,10.8) | 2.59 (2.06, 3.13) | 7.05 (6.55,7.54) | 8.33 (7.62, 9.05) | 1.28 (0.76, 1.81) |
| $\Delta$ 3 to 5 yrs. | -0.28(-0.33,-0.23) | -0.19 (-0.47,-0.09) | 0.08(-0.19,-0.37) | -0.60 (-0.87,-0.33) | -0.52 (-0.92,-0.13) | 0.07 (-0.21,0.36) |
| MD Age 5 yrs. | 7.11 (7.01, 7.22) | 9.89 (9.28, 10.5) | 2.77 (2.15, 3.40) | 5.84 (5.28, 6.40) | 7.28 (6.46, 8.09) | 1.43 (0.82, 2.05) |
| $\Delta$ 5 to 7 yrs. | 0.43 (0.27,0.58) | 1.46 (0.29,2.62) | 1.03 (-0.14,2.20) | 0.75 (-0.16,1.67) | 1.18 (-0.32,2.69) | 0.43 (-0.76, 1.63) |
| MD Age 7 yrs. | 7.98 (7.67, 8.29) | 12.82(10.52,15.1) | 4.83 (2.52,7.15) | 7.35 (5.55, 9.14) | 9.65 (6.70, 12.6) | 2.30 (-0.05,4.65) |
| $\Delta$ 7 to 9 yrs. | -0.13 (-0.19,-0.08) | -0.37 (-0.77,-0.02) | -2.33(-0.63,0.17) | -0.24 (-0.56,-0.07) | -0.34 (-0.86, 0.17) | -0.09(-0.51, 0.31) |
| MD Age 9 yrs. | 7.70 (7.47, 7.93) | 12.07(10.41,13.7) | 4.37 (2.68,6.05) | 6.85 (5.52, 8.18) | 8.96 (6.81, 11.1) | 2.10 (0.40, 3.81) |
|  | **Energy Poverty at 9 months only** | | | | | |
| MD Age 3 yrs. | 7.68 (7.59, 7.77) | 9.20 (8.89, 9.51) | 1.51 (1.18, 1.84) | 7.04 (6.55, 7.53) | 7.48 (6.91, 8.05) | 0.43 (0.11, 0.76) |
| $\Delta$ 3 to 5 yrs. | -0.28 (-0.33,-0.23) | -0.19 (-0.36, -0.03) | 0.08 (-0.08, -0.25) | -0.58 (-0.85,-0.32) | -0.47 (-0.79,-0.16) | 0.10 (-0.07, 0.28) |
| MD Age 5 yrs. | 7.11 (7.01, 7.22) | 8.80 (8.45, 9.16) | 1.68 (1.31, 2.05) | 5.87 (5.32, 6.42) | 6.52 (5.88, 7.17) | 0.64 (0.28, 1.01) |
| $\Delta$ 5 to 7 yrs. | 0.43 (0.27, 0.58) | 0.57 (-0.09, 1.24) | 0.14 (-0.54, -0.83) | 0.76 (-0.14, 1.66) | 0.44 (-0.68, 1.56) | -0.31 (-1.02, 0.38) |
| MD Age 7 yrs. | 7.98 (7.67, 8.29) | 9.95 (8.63,11.27) | 1.97 (0.62, 3.32) | 7.40 (5.63, 9.16) | 7.41 (5.21, 9.60) | 0.01 (-1.37, 1.39) |
| $\Delta$ 7 to 9 yrs. | -0.13 (-0.19,-0.08) | -0.13 (-0.36, -0.09) | 0.00 (-0.23,0.23) | -0.26 (-0.57, 0.05) | -0.15 (-0.54, 0.22) | 0.10 (-0.14, 0.34) |
| MD Age 9 yrs. | 7.70 (7.47, 7.93) | 9.67 (8.72,10.63) | 1.97 (0.98, 2.95) | 6.87 (5.56, 818) | 7.09 (5.48, 8.70) | 0.21 (-0.78, 1.22) |
|  | **Energy Poverty at 3 years only** | | | | | |
| MD Age 3 yrs. | 7.61 (7.52, 7.70) | 9.45 (9.17, 9.74) | 1.84 (1.54, 2.14) | 6.92 (6.43, 7.41) | 7.90 (7.35, 8.46) | 0.98 (0.68, 1.27) |
| $\Delta$ 3 to 5 yrs. | -0.28 (-0.32,-0.23) | -0.27 (-0.42, -0.12) | 0.00 (-0.15, 0.16) | -0.59 (-0.85,-0.32) | -0.57 (-0.88,-0.27) | 0.01 (-0.15, 0.17) |
| MD Age 5 yrs. | 7.05 (6.94, 7.15) | 8.90 (8.57, 9.22) | 1.84 (1.50, 2.19) | 5.74 (5.18, 6.30) | 6.75 (6.12, 7.38) | 1.00 (0.66, 1.34) |
| $\Delta$ 5 to 7 yrs. | 0.39 (0.23, 0.55) | 0.84 (0.24, 1.43) | 0.44 (-0.17, 1.06) | 0.57 (-0.34, 1.49) | 0.61 (-0.47, 1.70) | 0.03 (-0.60, 0.67) |
| MD Age 7 yrs. | 7.84 (7.53, 8.16) | 10.58 (9.40, 11.76) | 2.73 (1.51, 3.95) | 6.89 (5.10, 8.68) | 7.97 (5.85, 10.09) | 1.08 (-0.16, 2.32) |
| $\Delta$ 7 to 9 yrs. | -0.13 (-0.19,-0.08) | -0.16 (-0.37, 0.04) | -0.02 (-0.24, 0.18) | -0.20 (-0.52, 0.10) | -0.13 (-0.50, 0.24) | 0.07 (-0.14, 0.29) |
| MD Age 9 yrs. | 7.57 (7.33, 7.80) | 10.25 (9.39, 11.11) | 2.67 (1.78, 3.57) | 6.57 (5.15, 7.80) | 7.71 (6.15, 9.27) | 1.23 (0.32, 2.14) |
| **Adjusted for:** household income, household composition, household home owner, primary care giver (PCG) age, PCG education, PCG chronic illness and PCG depression. $MD mean difference in SDQ score , \Delta mean difference in change per year of SDQ score.$  **Abbreviations:** yrs. years of age, tSDQ total Strengths and Difficulties Questionnaire, EP Energy Poverty, CI 95% Confidence Interval. | | | | | | |

**Table S2:** Mean trajectories of total difficulties scores at 3, 5, 7 and 9 years of age by ‘Gone without Heat’ exposure at 9 months only, 3 years only and combined 9 months or 3 years of age using the Irish Growing Up in Ireland – Infant Cohort.

|  | **Unadjusted** | | | **Adjusted** | | |
| --- | --- | --- | --- | --- | --- | --- |
|  | **No EP** | **EP** | **No EP vs EP** | **No EP** | **EP** | **No EP vs EP** |
| **tSDQ** | **Mean trajectory (95% CI)** | **Mean trajectory (95% CI)** | **Mean difference in trajectory (95% CI)** | **Mean trajectory (95% CI)** | **Mean trajectory (95% CI)** | **Mean difference in trajectory comparing EP to no EP**  **(95% CI)** |
|  | **Gone without heat at 9 months** | | | | | |
| MD Age 3 yrs. | 7.77 (7.68, 7.86) | 10.22 (9.52, 10.92) | 2.45 (1.75, 3.16) | 7.07 (6.58, 7.56) | 8.06 (7.23, 8.89) | 0.99 (0.30, 1.68) |
| $\Delta$ 3 to 5 yrs. | -0.28 (-0.32 -0.23) | -0.47(-0.84,-0.09) | -0.19 (-0.57, 0.19) | -0.57(-0.84, -0.31) | -0.81(-1.27,-0.35) | -0.23 (-0.61, 0.15) |
| MD Age 5 yrs. | 7.22 (7.12, 7.32) | 9.29 (8.49, 10.09) | 2.07 (1.27, 2.88) | 5.92 (5.37, 6.47) | 6.45 (5.50, 7.39) | 0.52 (-0.26, 1.30) |
| $\Delta$ 5 to 7 yrs. | 0.43 (0.27, 0.58) | 1.45 (-0.14, 3.04) | 1.03 (-0.57, 2.63) | 0.74 (-0.17, 1.64) | 1.18 (-0.67, 3.03) | 0.45 (-1.18, 2.07) |
| MD Age 7 yrs. | 8.07 (7.77, 8.37) | 12.19 (9.06, 15.33) | 4.13 (0.97, 7.28) | 7.40 (5.63, 9.16) | 8.81 (5.19, 12.44) | 1.41 (-1.77, 4.60) |
| $\Delta$ 7 to 9 yrs. | -0.13 (-0.19 -0.08) | -0.45 (-0.99, 0.09) | -0.32 (-0.86, 0.23) | -0.25 (-0.57, 0.06) | -0.44 (-1.07, 0.19) | -0.19 (-0.74, 0.37) |
| MD Age 9 yrs. | 7.80 (7.57, 8.02) | 11.29 (9.03, 13.55) | 3.49 (1.22, 5.76) | 6.89 (5.58, 8.20) | 7.93 (5.32, 10.55) | 1.04 (-1.24, 3.32) |
|  | **Gone without heat at 3 years** | | | | | |
| MD Age 3 yrs. | 7.75 (7.66, 7.84) | 10.27 (9.61, 10.92) | 8.70 (7.91, 9.49) | 6.99 (6.50, 7.48) | 2.52 (1.85, 3.18) | 1.71 (1.07, 2.35) |
| $\Delta$ 3 to 5 yrs. | -0.28 (-0.32,-0.23) | -0.56 (-0.92,-0.21) | -0.88 (-1.31 -0.44) | -0.58 (-0.85,-0.32) | -0.29 (-0.64, 0.07) | -0.30 (-0.65, 0.06) |
| MD Age 5 yrs. | 7.19 (7.09, 7.29) | 9.14 (8.37, 9.90) | 6.95 (6.04, 7.86) | 5.83 (5.27, 6.38) | 1.94 (1.17, 2.72) | 1.12 (0.37, 1.87) |
| $\Delta$ 5 to 7 yrs. | 0.41 (0.26, 0.57) | 1.04 (-0.30, 2.38) | 0.60 (-1.01, 2.21) | 0.57 (-0.35, 1.49) | 0.63 (-0.72, 1.98) | 0.03 (-1.34, 1.40) |
| MD Age 7 yrs. | 8.02 (7.72, 8.33) | 11.22 (8.59, 13.85) | 8.15 (5.01, 11.29) | 6.97 (5.19, 8.76) | 3.20 (0.55, 5.84) | 1.18 (-1.49, 3.84) |
| $\Delta$ 7 to 9 yrs. | -0.13 (-0.19 -0.08) | -0.29 (-0.76, 0.18) | -0.19 (-0.75, 0.37) | -0.20 (-0.51, 0.12) | -0.16 (-0.63, 0.31) | 0.00 (-0.47, 0.48) |
| MD Age 9 yrs. | 7.75 (7.52, 7.98) | 10.64 (8.70, 12.57) | 7.76 (5.45, 10.07) | 6.58 (5.25, 7.90) | 2.88 (0.93, 4.83) | 1.18 (-0.77, 3.14) |
|  | **Gone without heat at 9 months or 3 years** | | | | | |
| MD Age 3 yrs. | 7.74 (7.65, 7.83) | 10.08 (9.58, 10.59) | 2.35 (1.83, 2.86) | 7.03 (6.54, 7.51) | 8.30 (7.62, 8.99) | 1.28 (0.78, 1.78) |
| $\Delta$ 3 to 5 yrs. | -0.27(-0.32,-0.22) | -0.51 (-0.79, -0.24) | -0.24 (-0.52,0.04) | -0.57 (-0.83,-0.30) | -0.82 (-1.20,-0.45) | -0.26 (-0.54,0.02) |
| MD Age 5 yrs. | 7.19 (7.09, 7.29) | 9.06 (8.47, 9.64) | 1.86 (1.27, 2.46) | 5.90 (5.35, 6.44) | 6.66 (5.88, 7.43) | 0.76 (0.18, 1.34) |
| $\Delta$ 5 to 7 yrs. | 0.42 (0.26, 0.57) | 1.19 (0.10, 2.28) | 0.77 (-0.33, 1.87) | 0.73 (-0.18, 1.64) | 0.93 (-0.48, 2.34) | 0.20 (-0.92, 1.32) |
| MD Age 7 yrs. | 8.03 (7.73, 8.34) | 11.44 (9.30, 13.58) | 3.41 (1.25, 5.57) | 7.36 (5.59, 9.12) | 8.52 (5.77, 11.28) | 1.17 (-1.03, 3.36) |
| $\Delta$ 7 to 9 yrs. | -0.13 (-0.19,-0.08) | -0.30 (-0.67, 0.08) | -0.16 (-0.54, 0.21) | -0.25 (-0.57, 0.06) | -0.28 (-0.77, 0.20) | -0.03 (-0.42, 0.36) |
| MD Age 9 yrs. | 7.76 (7.54, 7.99) | 10.84 (9.28, 12.40) | 3.08 (1.50, 4.65) | 6.85 (5.54, 8.16) | 7.96 (5.95, 9.97) | 1.11 (-0.48, 2.70) |
| **Adjusted for:** household income, household composition, household home owner, primary care giver (PCG) age, PCG education, PCG chronic illness and PCG depression. $MD mean difference in SDQ score , \Delta mean difference in change per year of SDQ score.$  **Abbreviations:** yrs. years of age, tSDQ total Strengths and Difficulties Questionnaire, EP Energy Poverty, CI 95% Confidence Interval. | | | | | | |

**Table S3:** Mean trajectories of total difficulties scores at 3, 5, 7 and 9 years of age by ‘Cold Home’ exposure at 9 months only, 3 years only and combined 9 months or 3 years of age using the Irish Growing Up in Ireland – Infant Cohort.

|  | **Unadjusted** | | | **Adjusted** | | |
| --- | --- | --- | --- | --- | --- | --- |
|  | **No EP** | **EP** | **No EP vs EP** | **No EP** | **EP** | **No EP vs EP** |
| **tSDQ** | **Mean trajectory (95% CI)** | **Mean trajectory (95% CI)** | **Mean difference in trajectory (95% CI)** | **Mean trajectory (95% CI)** | **Mean trajectory (95% CI)** | **Mean difference in trajectory comparing EP to no EP**  **(95% CI)** |
|  | **Cold home at 9 months** | | | | | |
| MD Age 3 yrs. | 7.71 (7.62, 7.80) | 9.13 (8.80, 9.46) | 1.42 (1.08, 1.77) | 7.06 (6.57, 7.55) | 7.43 (6.84, 8.01) | 0.36 (0.02, 0.70) |
| $\Delta$ 3 to 5 yrs. | -0.29 (-0.34 -0.24) | -0.14 (-0.32, 0.03) | 0.15 (-0.04, 0.33) | -0.59(-0.85, -0.32) | -0.41(-0.73,-0.10) | 0.17 (-0.01, 0.36) |
| MD Age 5 yrs. | 7.13 (7.03, 7.23) | 8.85 (8.47, 9.22) | 1.72 (1.33, 2.11) | 5.88 (5.34, 6.43) | 6.60 (5.94, 7.25) | 0.71 (0.33, 1.10) |
| $\Delta$ 5 to 7 yrs. | 0.43 (0.27, 0.59) | 0.60 (-0.10, 1.31) | 0.17 (-0.55, 0.90) | 0.76 (-0.15, 1.66) | 0.49 (-0.66, 1.63) | -0.27 (-1.01, 0.47) |
| MD Age 7 yrs. | 7.99 (7.69, 8.30) | 10.06 (8.67, 11.44) | 2.06 (0.64, 3.48) | 7.40 (5.63, 9.16) | 7.57 (5.33, 9.81) | 0.17 (-1.28, 1.62) |
| $\Delta$ 7 to 9 yrs. | -0.14 (-0.19 -0.08) | -0.17 (-0.41, 0.07) | -0.03 (-0.28, 0.21) | -0.26 (-0.57, 0.05) | -0.20 (-0.59, 0.20) | 0.06 (-0.19, 0.32) |
| MD Age 9 yrs. | 7.72 (7.49, 7.95) | 9.71 (8.71, 10.72) | 1.99 (0.96, 3.03) | 6.88 (5.57, 8.19) | 7.17 (5.53, 8.82) | 0.29 (-0.76, 1.34) |
|  | **Cold home at 3 years** | | | | | |
| MD Age 3 yrs. | 7.64 (7.55, 7.74) | 9.36 (9.07, 9.66) | 1.72 (1.41, 2.03) | 6.97 (6.48, 7.46) | 7.81 (7.24, 8.37) | 0.84 (0.53, 1.15) |
| $\Delta$ 3 to 5 yrs. | -0.29 (-0.33 -0.24) | -0.24 (-0.40, -0.08) | 0.05 (-0.12, 0.21) | -0.59 (-0.86 -0.33) | -0.54(-0.84,-0.23) | 0.06 (-0.11, 0.23) |
| MD Age 5 yrs. | 7.07 (6.97, 7.18) | 8.89 (8.55, 9.23) | 1.82 (1.46, 2.18) | 5.78 (5.22, 6.33) | 6.73 (6.09, 7.37) | 0.96 (0.60, 1.31) |
| $\Delta$ 5 to 7 yrs. | 0.39 (0.23, 0.55) | 0.97 (0.34, 1.60) | 0.58 (-0.07, 1.23) | 0.56 (-0.35, 1.48) | 0.76 (-0.35, 1.87) | 0.20 (-0.47, 0.86) |
| MD Age 7 yrs. | 7.85 (7.54, 8.17) | 10.83 (9.60, 12.07) | 2.98 (1.70, 4.26) | 6.90 (5.12, 8.69) | 8.25 (6.09, 10.42) | 1.35 (0.05, 2.66) |
| $\Delta$ 7 to 9 yrs. | -0.14 (-0.19 -0.08) | -0.19 (-0.40, 0.03) | -0.05(-0.27, 0.17) | -0.20 (-0.52, 0.11) | -0.16 (-0.54, 0.22) | 0.04 (-0.18, 0.27) |
| MD Age 9 yrs. | 7.58 (7.35, 7.82) | 10.46 (9.56, 11.36) | 2.88 (1.94, 3.81) | 6.49 (5.17, 7.82) | 7.94 (6.34, 9.53) | 1.44 (0.49, 2.39) |
|  | **Cold home at 9months or 3 years** | | | | | |
| MD Age 3 yrs. | 7.61 (7.52, 7.71) | 9.08 (8.84, 9.32) | 1.47 (1.21, 1.72) | 7.01 (6.52, 7.50) | 7.55 (7.01, 8.08) | 0.53 (0.27, 0.79) |
| $\Delta$ 3 to 5 yrs. | -0.29 (-0.34,-0.24) | -0.20 (-0.33,-0.07) | 0.09 (-0.05, 0.22) | -0.59(-0.86, -0.33) | -0.48 (-0.77,-0.19) | 0.11 (-0.03, 0.26) |
| MD Age 5 yrs. | 7.04 (6.93, 7.14) | 8.68 (8.41, 8.95) | 1.64 (1.35, 1.94) | 5.83 (5.28, 6.37) | 6.59 (5.98, 7.19) | 0.076 (0.47, 1.06) |
| $\Delta$ 5 to 7 yrs. | 0.41 (0.25, 0.58) | 0.72 (0.21, 1.22) | 0.30 (-0.23, 0.83) | 0.76 (-0.15, 1.67) | 0.66 (-0.38, 1.69) | -0.10 (-0.65, 0.45) |
| MD Age 7 yrs. | 7.87 (7.55, 8.18) | 10.11 (9.12, 11.11) | 2.25 (1.20, 3.29) | 7.34 (5.57, 9.11) | 7.90 (5.89, 9.91) | 0.56 (-0.52, 1.64) |
| $\Delta$ 7 to 9 yrs. | -0.14 (-0.19,-0.08) | -0.17 (-0.34, 0.01) | -0.03 (-0.21, 0.15) | -0.27 (-0.58, 0.05) | -0.20 (-0.56, 0.16) | 0.06 (-0.12, 0.25) |
| MD Age 9 yrs. | 7.59 (7.35, 7.83) | 9.78 (9.05, 10.50) | 2.19 (1.42, 2.95) | 6.81 (5.50, 8.12) | 7.50 (6.02, 8.98) | 0.69 (-0.09, 1.47) |
| **Adjusted for:** household income, household composition, household home owner, primary care giver (PCG) age, PCG education, PCG chronic illness and PCG depression. $MD mean difference in SDQ score , \Delta mean difference in change per year of SDQ score.$  **Abbreviations:** yrs. years of age, tSDQ total Strengths and Difficulties Questionnaire, EP Energy Poverty, CI 95% Confidence Interval. | | | | | | |

**Table S4:** Mean trajectories of total difficulties scores at 9, 13 and 17/18 years of age by ’Gone without Heat’ and ‘Cold Home’ exposure at 9 years of age using the Irish Growing Up in Ireland – Child Cohort.

|  | **Unadjusted** | | | **Adjusted** | | |
| --- | --- | --- | --- | --- | --- | --- |
|  | **No EP** | **EP** | **No EP vs EP** | **No EP** | **EP** | **No EP vs EP** |
| **tSDQ** | **Mean trajectory (95% CI)** | **Mean trajectory (95% CI)** | **Mean difference in trajectory (95% CI)** | **Mean trajectory (95% CI)** | **Mean trajectory (95% CI)** | **Mean difference in trajectory comparing EP to no EP**  **(95% CI)** |
|  | **Gone without heat at 9 years** | | | | | |
| MD Age 9 yrs. | 7.27 (7.17, 7.38) | 9.69 (9.22, 10.16) | 2.42 (1.94, 2.89) | 6.58 (6.07, 7.10) | 8.16 (7.49, 8.84) | 1.58 (1.12, 2.04) |
| $\Delta$ 9 to 13 yrs. | -0.42 (-0.49,-0.35) | -0.83 (-1.13,-0.52) | -0.41(-0.72,-0.09) | -0.72(-1.07,-0.37) | -1.12(-1.58,-0.66) | -0.40 (-0.72,-0.09) |
| MD Age 13 yrs. | 6.43 (6.29, 6.58) | 8.04 (7.39, 8.68) | 1.60 (0.94, 2.27) | 5.14 (4.42, 5.86) | 5.92 (4.96, 6.87) | 0.78 (0.12, 1.43) |
| $\Delta$ 13 to 17/18 yrs. | 0.02 (-0.01, 0.05) | -0.03 (-0.16, 0.10) | -0.04 (-0.18, 0.09) | 0.18 (0.04, 0.33) | 0.17 (-0.03, 0.36) | -0.02(-0.15, 0.11) |
| MD Age 17/18 yrs. | 6.47 (6.36, 6.58) | 7.99 (7.48, 8.49) | 1.52 (1.00, 2.03) | 5.51 (4.95, 6.07) | 6.25 (5.51, 6.99) | 0.74 (0.23, 1.24) |
|  | **Gone without heat at 9 years** | | | | | |
| MD Age 9 yrs. | 7.36 (7.25, 7.46) | 13.25 (11.96,14.54) | 5.89 (4.60, 7.19) | 6.61 (6.10, 7.13) | 11.13 (9.80,12.45) | 4.51 (3.27, 5.75) |
| $\Delta$ 9 to 13 yrs. | -0.43(-0.50,-0.37) | -1.80 (-2.62,-0.97) | -1.37 (-2.20,-0.54) | -0.73 (-1.07,-0.38) | -2.13 (-3.02,-1.24) | -1.41(-2.24,-0.58) |
| MD Age 13 yrs. | 6.49 (6.35, 6.63) | 9.65 (7.89, 11.41) | 3.16 (1.39, 4.93) | 5.16 (4.44, 5.88) | 6.86 (5.00, 8.72) | 1.70 (-0.03, 3.43) |
| $\Delta$ 13 to 17/18 yrs. | 0.02 (-0.01, 0.04) | 0.07 (-0.29, 0.43) | 0.05 (-0.31, 0.41) | 0.18 (0.04, 0.33) | 0.29 (-0.10, 0.67) | 0.10 (-0.25, 0.46) |
| MD Age 17/18 yrs. | 6.52 (6.41, 6.63) | 9.78 (8.41, 11.16) | 3.26 (1.88, 4.64) | 5.53 (4.97, 6.09) | 7.43 (6.00, 8.87) | 1.91 (0.57, 3.24) |
| **Adjusted for:** household income, household composition, household home owner, primary care giver (PCG) age, PCG education, PCG chronic illness and PCG depression. $MD mean difference in SDQ score , \Delta mean difference in change per year of SDQ score.$  **Abbreviations:** yrs. years of age, tSDQ total Strengths and Difficulties Questionnaire, EP Energy Poverty, CI 95% Confidence Interval. | | | | | | |

**Table S5:** Comparison of household and caregiver characteristics included in analysis (n=10 170) and excluded from analysis (n=964) using the Irish Growing Up in Ireland – Infant Cohort.

|  | | **Excluded**  **(n=964)** | | **Included**  **(n=10 170)** | |
| --- | --- | --- | --- | --- | --- |
|  | | **n** | **(%)** | **n** | **(%)** |
| **Gender** | Female | 451 | (46.8) | 5 004 | (49.2) |
| **4 Category**  **Household type** | 1 parent, 1 child | 83 | (8.6) | 507 | (5) |
|  | 1 parent, 2+ child | 115 | (11.9) | 654 | (6.4) |
|  | 2 parents, 1 child | 360 | (37.3) | 3 334 | (32.8) |
| 2 parents, 2+ child | | 406 | (42.1) | 5 675 | (55.8) |
| **Equivalised Household Income (quintile)** | 1st | 297 | (30.8) | 1 922 | (18.9) |
|  | 2^nd^ | 182 | (18.9) | 1 743 | (17.1) |
|  | 3^rd^ | 138 | (14.3) | 1 832 | (18) |
|  | 4^th^ | 129 | (13.4) | 2 075 | (20.4) |
|  | 5^th^ | 105 | (10.9) | 1 847 | (18.2) |
| **Accommodation** | House owner | 391 | (40.6) | 7 014 | (69) |
| **Rel. of PCG to child** | Parent | 963 | (99.9) | 10 169 | (99.9) |
| **PCG gender** | Female | 957 | (99.3) | 10 139 | (99.7) |
| **PCG age (years)** | <26 | 290 | (30.1) | 1 710 | (16.8) |
|  | 27-30 | 221 | (22.9) | 2 052 | (20.2) |
|  | 31-35 | 274 | (28.4) | 3 679 | (36.2) |
|  | 36-39 | 136 | (14.1) | 2 042 | (20.1) |
|  | 40+ | 43 | (4.5) | 687 | (6.8) |
| **PCG degree** | Yes | 289 | (30) | 3 742 | (36.8) |
| **PCG chronic health prob.** | Yes | 104 | (10.8) | 1 166 | (11.5) |
| **PCG depression** | Yes | 140 | (14.5) | 1 040 | (10.2) |
| **Abbreviations:** SDQ strengths and difficulties questionnaire, PCG Primary Care Giver, Rel. Relationship, Prob. Problem. | | | | | |

**Table S6**: Mean trajectories, sex stratification and interaction effects of total difficulties, externalising and internalising scores at 3, 5, 7 and 9 years of age by energy poverty prior to 3 years of age using the Irish Growing Up in Ireland – Infant Cohort.

|  | **Male Adjusted** | | | **Female Adjusted** | | | **Male vs Female** |
| --- | --- | --- | --- | --- | --- | --- | --- |
|  | **No EP** | **EP** | **No EP vs EP** | **No EP** | **EP** | **No EP vs EP** | **EP** |
| **SDQ** | **Mean trajectory (95% CI)** | **Mean trajectory (95% CI)** | **Mean difference in trajectory (95% CI)** | **Mean trajectory (95% CI)** | **Mean trajectory (95% CI)** | **Mean difference in trajectory comparing EP to no EP (95% CI)** | **Mean difference in trajectory comparing female vs male with EP**  **(95% CI)** |
|  | **Total SDQ Difficulties Score** | | | | | | |
| MD Age 3 yrs. | 6.96 (6.47, 7.45) | 7.62 (7.09, 8.15) | 0.66 (0.41, 0.91) | 7.75 (7.26, 8.24) | 8.41 (7.88, 8.95) | 1.45 (1.14, 1.75) | 0.79 (0.62, 0.96) |
| $\Delta$ 3 to 5 yrs. | -0.57 (-0.83,-0.30) | -0.62 (-0.92,-0.31) | -0.05 (-0.23, 0.13) | -0.46 (-0.73,-0.19) | -0.51 (-0.83,-0.19) | 0.05 (-0.17, 0.27) | 0.10 (0.01, 0.20) |
| MD Age 5 yrs. | 5.82 (5.27, 6.37) | 6.38 (5.75, 7.01) | 0.56 (0.93, 0.18) | 6.61 (6.04, 7.17) | 7.79 (7.16, 8.43) | 1.18 (0.80, 1.56) | 1.42 (0.97, 1.87) |
| $\Delta$ 5 to 7 yrs. | 0.70 (-0.21, 1.61) | 1.09 (-0.01, 2.21) | 0.39 (-0.34, 1.14) | 1.08 (0.17, 2.00 | 1.48 (0.31, 2.65) | 0.78 (-0.09, 1.66) | 0.39 (0.06, 0.71) |
| MD Age 7 yrs. | 7.22 (5.45, 8.99) | 8.58 (6.40, 10.75) | 1.35 (-0.09, 2.81) | 8.01 (6.23, 9.78) | 8.22 (6.04, 10.40) | 0.21 (-1.20, 1.63) | -0.36 (-2.24, 1.53) |
| $\Delta$ 7 to 9 yrs. | -0.24 (-0.56, 0.06) | -0.35 (-0.73, 0.03) | -0.10 (-0.35, 0.15) | -0.27 (-0.59, 0.04) | -0.37 (-0.77, 0.26) | -0.12 (-0.42, 0.17) | -0.03 (-0.14, 0.09) |
| MD Age 9 yrs. | 6.75 (5.41, 8.03) | 7.88 (6.28, 9.47) | 1.15 (0.09, 2.21) | 7.51 (6.19, 8.83) | 8.37 (6.77, 9.97) | 0.86 (-0.16, 1.89) | 0.50 (-0.86, 1.85) |
|  | **SDQ Externalising Score** | | | | | | |
| MD Age 3 yrs. | 4.58 (4.23, 4.93) | 5.00 (4.62, 5.37) | 0.41 (0.24, 0.59) | 5.20 (4.85, 5.55) | 5.61 (5.24, 5.99) | 1.03 (0.82, 1.25) | 0.62 (0.50, 0.74) |
| $\Delta$ 3 to 5 yrs. | -0.47 (-0.65,-0.28) | -0.45 (-0.66,-0.24) | 0.02 (-0.11, 0.15) | -0.31 (-0.50,-0.13) | -0.30 (-0.52,-0.07) | 0.17 (0.01, 0.33) | 0.15 (0.09, 0.22) |
| MD Age 5 yrs. | 3.65 (3.26, 4.04) | 4.10 (3.65, 4.54) | 0.45 (0.19, 0.71) | 4.27 (3.87, 4.66) | 5.30 (4.85, 5.74) | 1.03 (0.77, 1.3) | 1.20 (0.89, 1.52) |
| $\Delta$ 5 to 7 yrs. | 0.29 (-0.33, 0.91) | 0.40 (-0.36, 1.16) | 0.11 (-0.40, 0.61) | 0.57 (-0.05, 1.19) | 0.68 (-0.11, 1.47) | 0.39 (-0.21, 0.98) | 0.28 (0.06, 0.50) |
| MD Age 7 yrs. | 4.23 (3.04, 5.43) | 4.89 (3.42, 6.37) | 0.66 (-0.33, 1.65) | 4.85 (3.65, 6.05) | 4.80 (3.33 (6.28) | -0.05 (-1.01, 0.91) | -0.09 (-1.37, 1.19) |
| $\Delta$ 7 to 9 yrs. | -0.24 (-0.46,-0.02) | -0.27 (-0.54,0.00) | -0.03 (-0.20, 0.15) | -023 (-0.45, 0.01) | -0.26 (-0.54,0.02) | -0.01 (0.22, 0.20) | 0.01 (-0.07, 0.09) |
| MD Age 9 yrs. | 3.75 (2.88, 4.61) | 4.36 (3.30, 5.41) | 0.61 (-0.09, 1.31) | 4.36 (3.49, 5.24) | 4.88 (3.82, 5.94) | 0.51 (-0.17, 1.20) | 0.52 (-0.38, 1.420 |
|  | **SDQ Internalising Score** | | | | | | |
| MD Age 3 yrs. | 2.38 (2.13, 2.63) | 2.63 (2.36, 2.90) | 0.25 (0.12, 0.38) | 2.56 (2.30, 2.81) | 2.81 (2.53, 3.08) | 0.42 (0.27, 0.58) | 0.17 (0.09, 0.26) |
| $\Delta$ 3 to 5 yrs. | -0.11 (-0.26, 0.05) | -0.17 (-0.35, 0.00) | -0.07 (-0.17, 0.03) | -0.16 (-0.31, 0.00) | -0.22 (-0.41,-0.04) | -0.12 (-0.24, 0.01) | -0.05 (-0.10, 0.01) |
| MD Age 5 yrs. | 2.17 (1.89, 2.45) | 2.28 (1.96, 2.61) | 0.11 (-0.08, 0.31) | 2.34 (2.05, 2.63) | 2.51 (2.19, 2.84) | 0.17 (-0.03, 0.37) | 0.23 (-0.01, 0.47) |
| $\Delta$ 5 to 7 yrs. | 0.39 (-0.15, 0.93 | 0.68 (0.03, 1.34) | 0.29 (-0.15, 0.73) | 0.49 (-0.05, 1.03) | 0.78 (0.09, 1.47) | 0.39 (-0.13, 0.91) | 0.10 (-0.09, 0.29) |
| MD Age 7 yrs. | 2.95 (1.92, 3.99) | 3.65 (2.38, 4.92) | 0.70 (-0.16, 1.55) | 3.13 (2.09, 4.16) | 3.35 (2.07, 4.62) | 0.22 (-0.61, 1.05) | -0.30 (-1.41, 0.80) |
| $\Delta$ 7 to 9 yrs. | 0.00 (-0.18, 0.19) | -0.07 (-0.30, 0.15) | -0.08 (-0.23, 0.07) | -0.03 (-0.22, 0.15) | -0.11 (-0.34, 0.13) | -0.11 (-0.29, 0.07) | -0.03 (-0.10, 0.03) |
| MD Age 9 yrs. | 2.96 (2.20, 3.73) | 3.51 (2.57, 4.44) | 0.55 (-0.07, 1.17) | 3.13 (2.37, 3.90) | 3.46 (2.53, 4.40) | 0.33 (-0.27, 0.93) | -0.04 (-0.84, 0.76) |
| **Adjusted for:** household income, household composition, household home owner, primary care giver (PCG) age, PCG education, PCG chronic illness and PCG depression. $MD mean difference in SDQ score , \Delta mean difference in change per year of SDQ score.$  **Abbreviations:** yrs. years of age, SDQ Strengths and Difficulties Questionnaire, EP Energy Poverty, CI 95% Confidence Interval, Int Interaction. | | | | | | | |

**Table S7:** Mean trajectories, sex stratification and interaction effects of total difficulties, externalising and internalising scores at 9, 13 and 17/18 years of age by energy poverty at 9 years of age using the Irish Growing Up in Ireland – Child Cohort.

|  | **Male Adjusted** | | | **Female Adjusted** | | | **Male vs Female** |
| --- | --- | --- | --- | --- | --- | --- | --- |
|  | **No EP** | **EP** | **No EP vs EP** | **No EP** | **EP** | **No EP vs EP** | **EP** |
| **SDQ** | **Mean trajectory (95% CI)** | **Mean trajectory (95% CI)** | **Mean difference in trajectory comparing EP to no EP (95% CI)** | **Mean trajectory (95% CI)** | **Mean trajectory (95% CI)** | **Mean difference in trajectory comparing EP to no EP (95% CI)** | **Mean difference in trajectory comparing female vs male with EP**  **(95% CI)** |
|  | **Total SDQ Difficulties Score** | | | | | | |
| MD Age 9 yrs. | 6.56 (6.05, 7.08) | 8.30 (7.63, 8.96) | 1.73 (1.28, 2.19) | 7.27 (6.75, 7.78) | 9.00 (8.33, 9.67) | 2.44 (1.94, 2.93) | 0.90 (0.77, 1.03) |
| $\Delta$ 9 to 13 yrs. | -0.72 (-1.07,-0.38) | -1.03 (-1.55,-0.52) | -0.31 (-0.71, 0.09) | -0.52 (-0.86,-0.17) | -0.83 (-1.36,-0.30) | -0.10 (-0.55, 0.34) | 0.02 (-0.06, 0.11) |
| MD Age 13 yrs. | 5.12 (4.39, 5.84) | 6.23 (5.17, 7.30) | 1.12 (0.29, 1.94) | 5.82 (5.09, 6.56) | 6.42 (5.30, 7.54) | 0.60 (-0.30, 1.50) | 0.43 (-0.30, 1.16) |
| $\Delta$ 13 to 17/18 yrs. | 0.19 (0.04, 0.33) | 0.13 (-0.10, 0.35) | -0.06 (-0.24, 0.12) | -0.05 (-0.20, 0.09) | -0.11 (-0.34, 0.12) | -0.30 (-0.49,-0.11) | -0.05 (-0.09, -0.02) |
| MD Age 17/18 yrs. | 5.49 (4.93, 6.05) | 6.49 (5.68, 7.30) | 1.00 (0.38, 1.62) | 6.19 (5.62, 6.77) | 6.43 (5.59, 7.28) | 0.24 (-0.43, 0.91) | 0.48 (-0.04, 1.00) |
|  | **SDQ Externalising Score** | | | | | | |
| Md Age 9 yrs. | 3.71 (3.37, 4.04) | 4.34 (3.91, 4.78) | 0.64 (0.34, 0.93) | 4.60 (4.27, 4.94) | 5.24 (4.81, 5.68) | 1.53 (1.21, 1.86) | 0.81 (0.68, 0.95) |
| $\Delta$ 9 to 13 yrs. | -0.33 (-0.55,-0.11) | -0.29 (-0.62, 0.04) | 0.04 (-0.22, 0.30 | -0.31 (-0.53,-0.09) | -0.27 (-0.61, 0.07) | 0.06 (-0.22, 0.35) | 0.02 (-0.06, 0.11) |
| MD Age 13 yrs. | 3.04 (2.58, 3.51) | 3.76 (3.07, 4.44) | 0.72 (0.18, 1.25) | 3.94 (3.47, 4.41) | 4.19 (3.47, 4.91) | 0.25 (-0.33, 0.83) | 0.23 (-0.50, 0.96) |
| $\Delta$ 13 to 17/18 yrs. | -0.05 (-0.14, 0.04) | -0.14 (-0.28, 0.00) | -0.09 (-0.20, 0.02) | -0.10 (-0.19,-0.01) | -0.19 (-0.34,-0.05) | -0.14 (-0.26,-0.02) | -0.04 (-0.08, -0.01) |
| MD Age 17/18 yrs. | 2.94 (2.58, 3.30) | 3.47 (2.95, 3.99) | 0.53 (0.13, 0.93) | 3.84 (3.47, 4.21) | 3.95 (3.41, 4.50) | 0.12 (-0.32, 0.55) | 0.30 (-0.23, 0.82) |
|  | **SDQ Internalising Score** | | | | | | |
| MD Age 9 yrs. | 2.86 (2.56, 3.15) | 3.95 (3.57, 4.33) | 1.09 (0.83, 1.35) | 2.66 (2.37, 2.96) | 3.75 (3.37, 4.14) | 0.90 (0.61, 1.18) | -0.19 (-0.31, -0.08) |
| $\Delta$ 9 to 13 yrs. | -0.39 (-0.61,-0.17) | -0.74 (-1.07,-0.42) | -0.36 (-0.61,-0.10) | -0.20 (-0.43, 0.02) | -0.56 (-0.90,-0.22) | -0.17 (-0.45, 0.11) | 0.18 (0.10, 0.27) |
| MD Age 13 yrs. | 2.08 (1.64, 2.52) | 2.46 (1.81, 3.11) | 0.38 (-0.13, 0.89) | 1.89 (1.44, 2.33) | 2.25 (1.57, 2.93) | 0.36 (-0.19, 0.92) | -0.21 (-0.92, 0.50) |
| $\Delta$ 13 to 17/18 yrs. | 0.24 (0.14, 0.33) | 0.27 (0.13, 0.42) | 0.03 (-0.08, 0.15) | 0.05 (-0.05, 0.14) | 0.08 (-0.07, 0.23) | -0.16 (-0.28,-0.03) | -0.19 (-0.23, -0.16) |
| MD Age 17/18 yrs. | 2.56 (2.23, 2.88) | 3.00 (2.53, 3.48) | 0.45 (0.08, 0.81) | 2.36 (2.03, 2.69) | 2.49 (2.00, 2.99) | 0.13 (-0.27, 0.53) | -0.51 (-1.01, -0.01) |
| **Adjusted for:** household income, household composition, household home owner, primary care giver (PCG) age, PCG education, PCG chronic illness and PCG depression. $MD mean difference in SDQ score , \Delta mean difference in change per year of SDQ score.$  **Abbreviations:** yrs. years of age, SDQ Strengths and Difficulties Questionnaire, EP Energy Poverty, CI 95% Confidence Interval, Int Interaction. | | | | | | | |

**Table S8:** Description of outcome (strengths and difficulties questionnaire) included at each age and the number of outcomes used in analysis per participant using the Irish Growing Up in Ireland – Infant Cohort (n=11 134)

|  | **Included** | | **Missing** | |
| --- | --- | --- | --- | --- |
|  | **n** | **(%)** | **n** | **(%)** |
| **No. of SDQ results at each age:** | | |  |  |
| 3 years | 9 786 | (87.9) | 1 348 | (12.1) |
| 5 years | 8 996 | (80.8) | 2 138 | (19.2) |
| 7 years | 5 275 | (47.4) | 5 859 | (52.6) |
| 9 years | 8 022 | (72.1) | 3 112 | (27.9) |
|  |  |  |  |  |
| **No. of participants with:** |  |  |  |  |
|  | **n** | **(%)** |  |  |
| 0 SDQ results | 964 | (8.7) |  |  |
| 1 SDQ result | 943 | (8.5) |  |  |
| 2 SDQ results | 1 275 | (11.5) |  |  |
| 3 SDQ results | 3 222 | (28.9) |  |  |
| 4 SDQ results | 4 730 | (42.5) |  |  |
| Mean SDQ results (SD) | 2.9 | (1.3) |  |  |
| Abbreviations: n number, No. number, SDQ strengths and difficulties questionnaire, SD standard deviation. | | | | |

|  | **Included** | | **Missing** | |
| --- | --- | --- | --- | --- |
|  | **n** | **(%)** | **n** | **(%)** |
| **No. of SDQ results at each age:** | | |  |  |
| 9 years | 8518 | (99.4) | 50 | (0.6) |
| 13 years | 7 523 | (87.8) | 1 045 | (12.2) |
| 17/18 years | 6154 | (71.8) | 2 414 | (28.2) |
|  |  |  |  |  |
| **No. of participants with:** |  |  |  |  |
|  | **n** | **(%)** |  |  |
| 0 SDQ results | 8 | (0.1) |  |  |
| 1 SDQ result | 883 | (10.3) |  |  |
| 2 SDQ results | 1 719 | (20.1) |  |  |
| 3 SDQ results | 5 958 | (69.5) |  |  |
| Mean SDQ results (SD) | 2.6 | (0.7) |  |  |
| **Abbreviations:** n number, No. number, SDQ strengths and difficulties questionnaire, SD standard deviation | | | | |

**Table S9:** Description of outcome (strengths and difficulties questionnaire) included at each age and the number of outcomes used in analysis per participant using the Irish Growing Up in Ireland – Child Cohort (n=8 568)

**Table S10:** Mean trajectories of total difficulties scores from 3 to 9 years of age by energy poverty prior to 3 years of age using the Irish Growing Up in Ireland – Infant Cohort (n=4 730), including participants with an outcome measure at each wave.

|  | **Unadjusted** | | | **Adjusted** | | |
| --- | --- | --- | --- | --- | --- | --- |
|  | **No EP** | **EP** | **No EP vs EP** | **No EP** | **EP** | **No EP vs EP** |
| **SDQ** | **Mean trajectory (95% CI)** | **Mean trajectory (95% CI)** | **Mean difference in trajectory (95% CI)** | **Mean trajectory (95% CI)** | **Mean trajectory (95% CI)** | **Mean difference in trajectory comparing EP to no EP (95% CI)** |
|  | **Total SDQ Difficulties Score** | | | | | |
| MD Age 3 yrs. | 7.20 (7.1, 7.3) | 8.24 (7.86, 8.62) | 1.05 (0.64, 1.45) | 6.52 (5.83, 7.20) | 6.84 (6.07, 7.61) | 0.32 (-0.08, 0.72) |
| $\Delta$ 3 to 5 yrs. | -0.26 (-0.32,-0.20) | -0.09 (-0.28, 0.10) | 0.17 (-0.3, 0.37) | -0.46 (-0.82, 0.09) | -0.28 (-0.68, 0.12) | 0.18 (-0.03, 0.39) |
| MD Age 5 yrs. | 6.66 (6.53, 6.80) | 8.05 (7.63, 8.47) | 1.39 (0.95, 1.83) | 5.60 (4.85, 6.35) | 6.28 (5.44, 7.12) | 0.68 (0.24, 1.12) |
| $\Delta$ 5 to 7 yrs. | 0.38 (0.21, 0.55) | 0.65 (0.13, 1.17) | 0.27 (-0.27, 0.81) | 0.65 (-0.30, 1.61) | 0.55 (-0.51, 1.62) | -0.10 (-0.67, 0.46) |
| MD Age 7 yrs. | 7.43 (7.10, 7.76) | 9.37 (8.37, 10.37) | 1.93 (0.88, 2.98) | 6.91 (5.07, 8.75) | 7.39 (5.33, 9.44) | 0.47 (-0.61, 1.56) |
| $\Delta$ 7 to 9 yrs. | -0.15 (-0.21, 0.09) | -0.18 (-0.36,-0.01) | -0.03 (-0.21, 0.15) | -0.22 (-0.54, 0.10) | -0.16 (-0.53, 0.20) | 0.06 (-0.14, 0.25) |
| MD Age 9 yrs. | 7.12 (6.86, 7.38) | 8.99 (8.20, 9.78) | 1.87 (1.04, 2.70) | 6.47 (5.03, 7.91) | 7.06 (5.44, 8.67) | 0.59 (-0.26, 1.44) |
| **Adjusted for:** household income, household composition, household home owner, primary care giver (PCG) age, PCG education, PCG chronic illness and PCG depression. $MD mean difference in SDQ score , \Delta mean difference in change per year of SDQ score.$  **Abbreviations:** yrs. years of age, SDQ Strengths and Difficulties Questionnaire, EP Energy Poverty, CI 95% Confidence Interval | | | | | | |

**Table S11:** Mean trajectories of total difficulties scores from 9 to 17/18 years of age by energy poverty prior to 9 years of age using the Irish Growing Up in Ireland – Child Cohort (n= 5 958), including participants with an outcome measure at each wave.

|  | **Unadjusted** | | | **Adjusted** | | |
| --- | --- | --- | --- | --- | --- | --- |
|  | **No EP** | **EP** | **No EP vs EP** | **No EP** | **EP** | **No EP vs EP** |
| **SDQ** | **Mean trajectory (95% CI)** | **Mean trajectory (95% CI)** | **Mean difference in trajectory (95% CI)** | **Mean trajectory (95% CI)** | **Mean trajectory (95% CI)** | **Mean difference in trajectory comparing EP to no EP (95% CI)** |
|  | **Total SDQ Difficulties Score** | | | | | |
| MD Age 9 yrs. | 7.12 (6.99,7.24) | 9.66 (9.09, 10.22) | 2.54 (1.97, 3.12) | 6.58 (5.95, 7.21) | 8.38 (7.56, 9.21) | 1.81 (1.25, 2.36) |
| $\Delta$ 9 to 13 yrs. | -0.43 (-0.50,-0.35) | -1.05 (-1.38,-0.72) | -0.62 (-0.96,-0.28) | -0.75 (-1.13,0.36) | -1.38 (-1.89,-0.87) | -0.63 (-0.97,-0.29) |
| MD Age 13 yrs. | 6.26 (6.10, 6.42) | 7.56 (6.85, 8.27) | 1.30 (0.57, 2.03) | 5.08 (4.27, 5.89) | 5.63 (4.56, 6.68) | 0.54 (-0.17, 1.26) |
| $\Delta$ 13 to 17/18 yrs. | 0.02 (-0.01, 0.05) | -0.00 (-0.14,0.13) | -0.02 (-0.16, 0.11) | 0.18 (0.03, 0.33) | 0.18 (-0.02,0.38) | -0.00 (-0.14, 0.13) |
| MD Age 17/18 yrs. | 6.31 (6.18, 6.43) | 7.56 (6.99, 8.12) | 1.25 (0.67, 1.83) | 5.44 (4.80, 6.08) | 5.98 (5.14, 6.82) | 0.54 (-0.03, 1.11) |
| **Adjusted for:** household income, household composition, household home owner, primary care giver (PCG) age, PCG education, PCG chronic illness and PCG depression. $MD mean difference in SDQ score , \Delta mean difference in change per year of SDQ score.$  **Abbreviations:** yrs. years of age, SDQ Strengths and Difficulties Questionnaire, EP Energy Poverty, CI 95% Confidence Interval. | | | | | | |

**Figures**

**
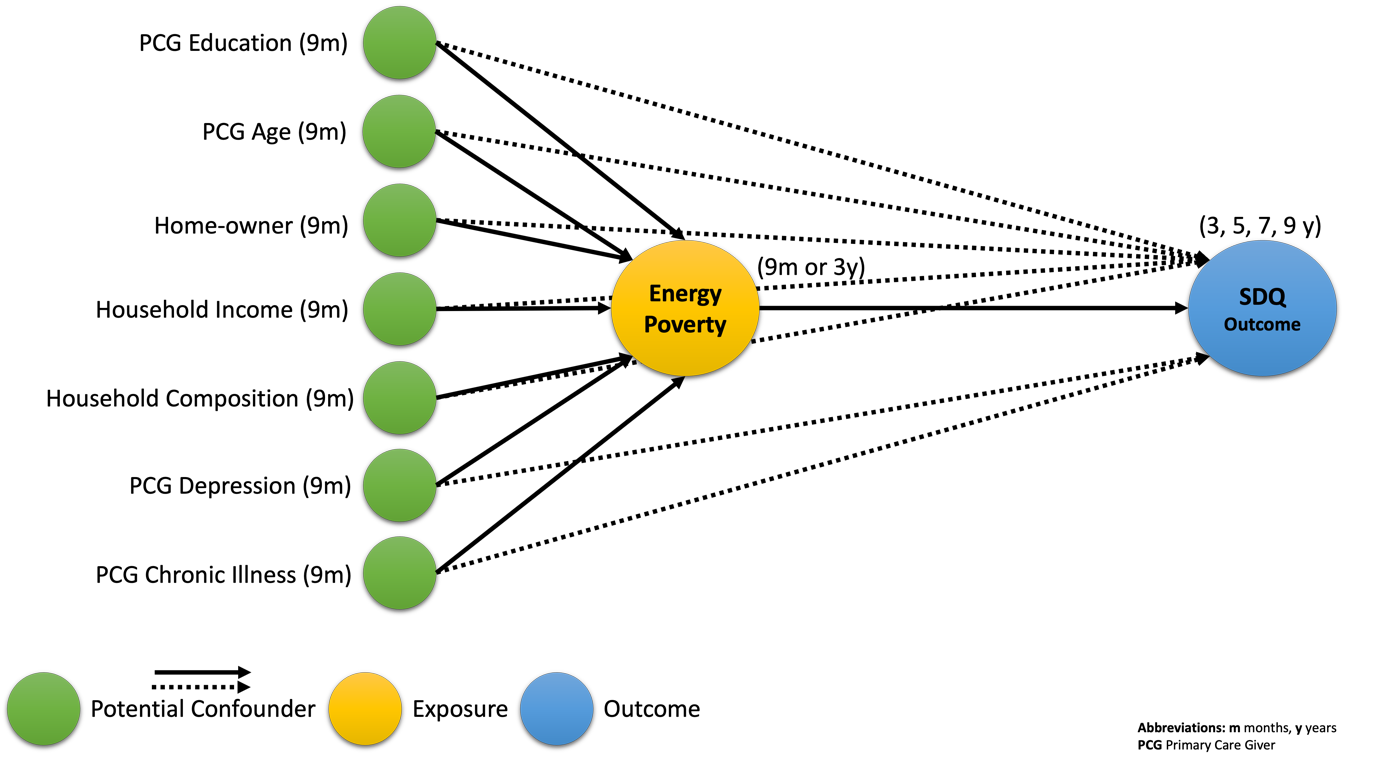
**

**Figure S1**: Directed acyclic graph (DAG) of study design using Irish Growing Up in Ireland Infant Cohort.


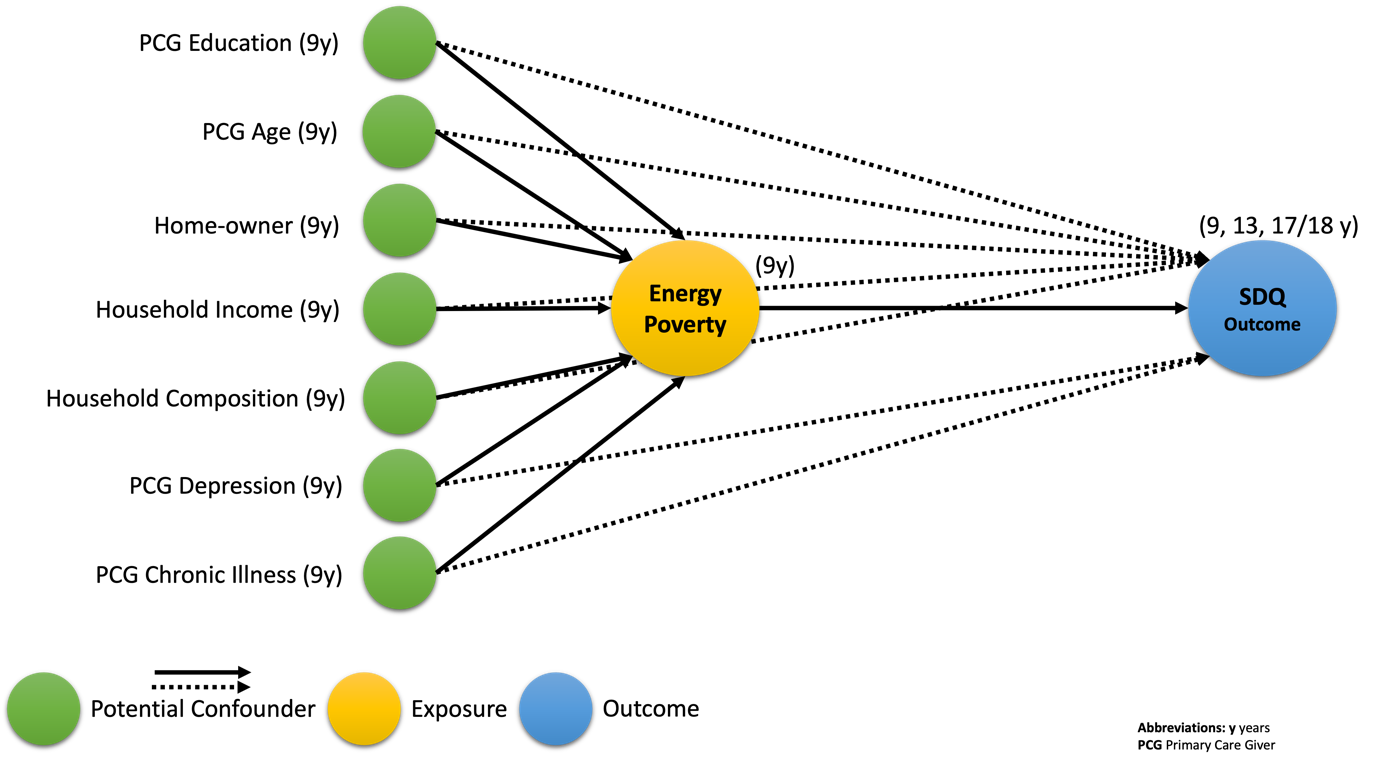


**Figure S2**: Directed acyclic graph (DAG) of study design using Irish Growing Up in Ireland Child Cohort.


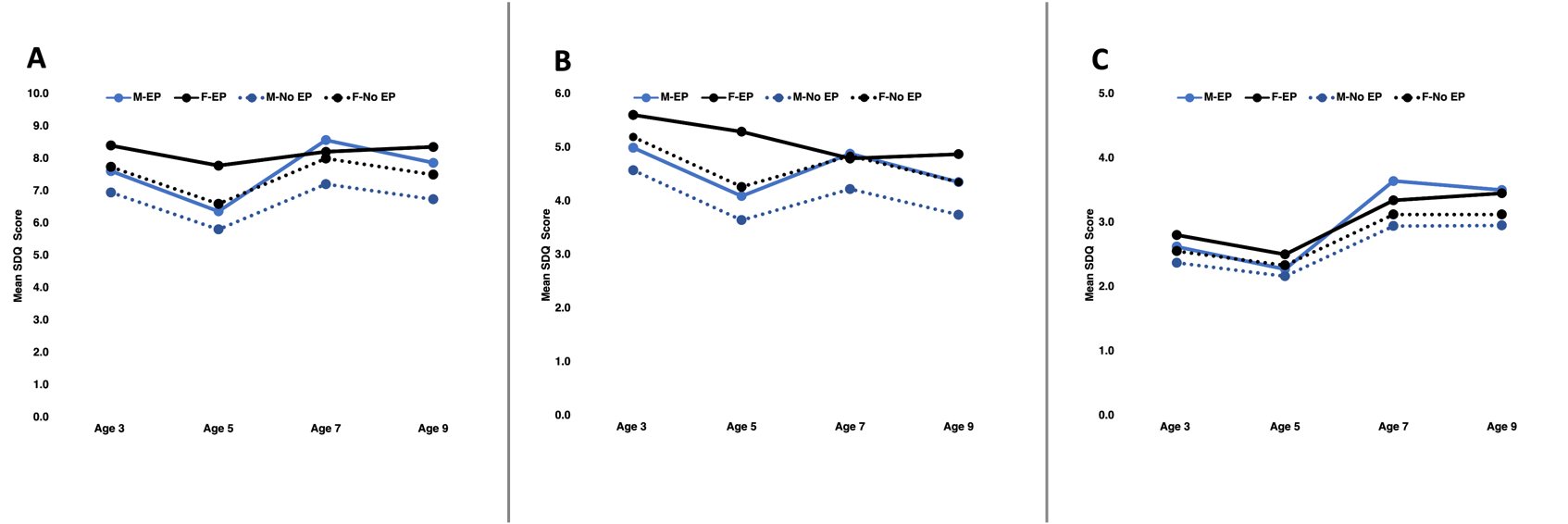


**Figure S3:** Trajectories of (A) total difficulties score, (B) externalising difficulties score, and (C) internalising difficulties score from 3 years to 9 years by energy poverty exposure in males (M-EP) and females (F-EP) prior to 3 years of age, from adjusted analysis (primary care giver age, primary care giver education, household composition, household income, home ownership, primary care giver chronic health status and depression status using the Growing Up in Ireland – Infant Cohort.

**Figure S4:** Trajectories of (A) total difficulties score, (B) externalising difficulties score, and (C) internalising difficulties score from 9 years to 17/18 years by energy poverty exposure in males (M-EP) and females (F-EP) at 9 years, from adjusted analysis (primary care giver age, primary care giver education, household composition, household income, home ownership, primary care giver chronic health status and depression status using the Growing Up in Ireland – Child Cohort.


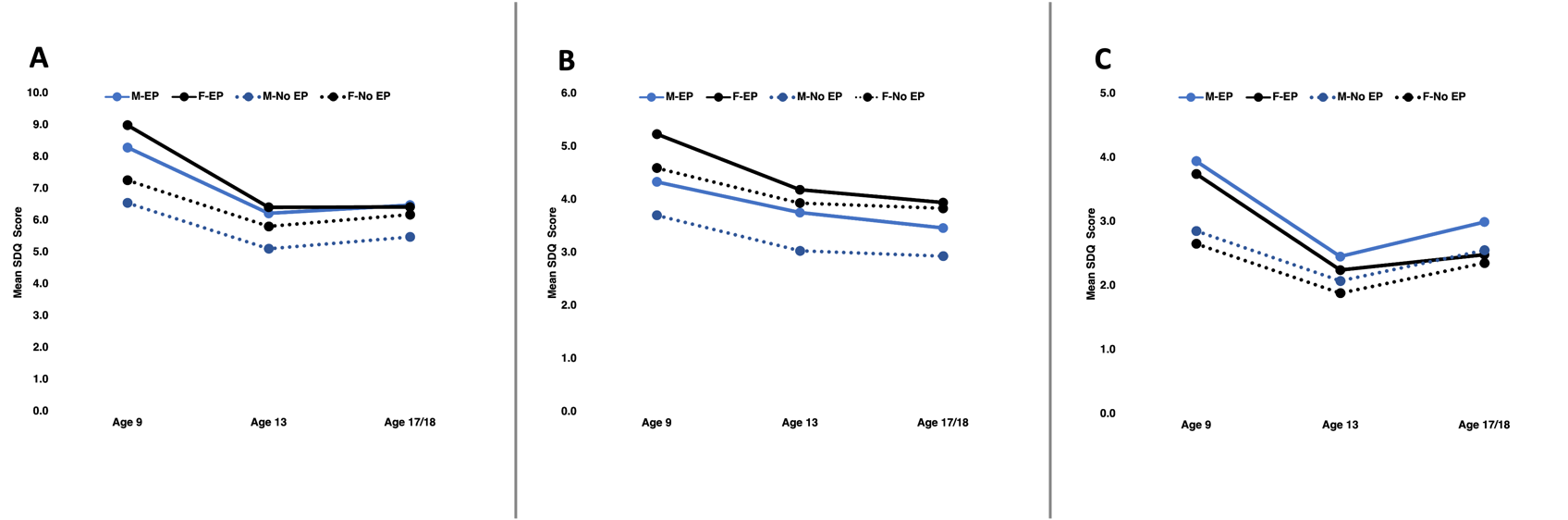

Supplement: Supplementary file 1 — Supplementary file1 (DOCX 4631 KB) [file 127_2024_2616_MOESM1_ESM.docx]
