# Supplementary material for: Household energy poverty and trajectories of emotional and behavioural difficulties in children and adolescents: findings from two prospective cohort studies
Source: Soc Psychiatry Psychiatr Epidemiol. 2024 Feb 12;59(8):1299–309. doi: 10.1007/s00127-024-02616-2 (PMC11291537; doi:10.1007/s00127-024-02616-2)
Supplement: Supplementary file 2 — Supplementary file2 (DOCX 19 KB) [file 127_2024_2616_MOESM2_ESM.docx]

**Supplementary 2**

**Sample code for implementing linear spline multilevel models**

It is possible to utilise the Stata Package ‘runnlwin’ to limit the amount of syntax required (please see (O’Keeffe et al., 2023)).

The code below assumes the data is in long format and this is the code that applies for energy poverty (EP1) and total strengths and difficulties (SDQ) at 3, 5, 7, and 9 years.

****# Generate the spline variable**

* sp1: SDQ from 3 years to 5 years.

* sp2: SDQ from 5 years to 7 years.

* sp3: SDQ from 7 years to 9 years.

🡪 mkspline sp1 2 sp2 3 sp3 = age_lw

****# Generate interactions**

🡪 gen EP1_sp1 = EP1*sp1

🡪 gen EP1_sp2 = EP1*sp2

🡪 gen EP1_sp3 = EP1*sp3

****# Crude model**

🡪 xtmixed SDQ EP1 sp1 sp2 sp3 EP1_sp1 EP1_sp2 EP1_sp3 || ID: sp1 sp2 sp3

****#Identifying the mean at each time point**

**** Reference group (i.e., no EP1)***

*mean outcome in the reference group at time 1 (i.e., no EP1)

🡪 lincom _cons

*mean rate of change per year in the reference group from 3 to 5 years (i.e., no EP1)

🡪 lincom sp1

**mean rate of change per year in the reference group from 5 to 7 years (i.e., no EP1)*

🡪 lincom sp2

**mean rate of change per year in the reference group from 7 to 9 years (i.e., no EP1)*

🡪 lincom sp3

***Exposed group (i.e., yes EP1)***

**mean outcome in people with any energy poverty at time 1*

🡪 lincom _cons + EP1

**mean rate of change per year in people with any energy from 3 to 5 years*

🡪 lincom sp1 + EP1_sp1

**mean rate of change per year in people with any energy poverty from 5 to 7 years*

🡪 lincom sp2 + EP1_sp2

**mean rate of change per year in people with any energy poverty from 7 to 9 years*

🡪 lincom sp3 + EP1_sp3

***Mean difference in rate of change per year***

**mean difference in outcome in people with any energy poverty at 3 years compared with reference category (i.e., no EP1).*

🡪 lincom EP1

**mean difference in rate of change per year in people with any energy from 3 to 5 years compared with reference category (i.e., no EP1).*

🡪 lincom EP1_sp1

**mean difference in rate of change per year in people with any energy poverty from 5 to 7 years compared with reference category (i.e., no EP1).*

🡪 lincom EP1_sp2

**mean difference in rate of change per year in people with any energy poverty from 7 to 9 years compared with reference category (i.e., no EP1).*

🡪 lincom EP1_sp3

**Mean at each age: 3 years**

**mean SDQ at age 3 in reference group (i.e., no EP1)*

🡪 lincom _cons

**mean SDQ at age 3 in those with EP1*

🡪 lincom _cons + EP1

**mean difference in SDQ at age 3 comparing EP to reference (i.e., no EP1)*

🡪 lincom EP1

**Mean at each age: 5 years**

**mean SDQ at age 5 in reference group (i.e., no EP1)*

🡪 lincom _cons + sp1*2

**mean SDQ at age 5 in those with EP1*

🡪 lincom _cons + EP1 + (sp1 + EP1_sp1)*2

**mean difference in SDQ at age 5 comparing EP1 to reference (i.e., no EP1)*

🡪 lincom (_cons + EP1 + (sp1 + EP1_sp1)*2) - (_cons + sp1*2)

**Mean at each age: 7 years**

**mean SDQ at age 7 in reference group (i.e., no EP1)*

🡪 lincom _cons + sp1*2 + sp2*2

**mean SDQ at age 7 in those with EP1*

🡪 lincom _cons + EP1 + (sp1 + EP1_sp1)*2 + (sp2 + EP1_sp2)*2

**mean difference in SDQ at age 7 comparing EP1 to reference (i.e., no EP1)*

🡪 lincom (_cons + EP1 + (sp1 + EP1_sp1)*2 + (sp2 + EP1_sp2)*2) - (_cons + sp1*2 + sp2*2)

**Mean at each age: 9 years**

**mean SDQ at age 9 in reference group (i.e., no EP1)*

🡪 lincom _cons + sp1*2 + sp2*2 + sp3*2

**mean SDQ at age 9 in those with EP1*

🡪 lincom _cons + EP1 + (sp1 + EP1_sp1)*2 + (sp2 + EP1_sp2)*2 + (sp3 + EP1_sp3)*2

**mean difference in SDQ at age 9 comparing EP1 to reference (i.e., no EP1)*

🡪 lincom (_cons + EP1 + (sp1 + EP1_sp1)*2 + (sp2 + EP1_sp2)*2 + (sp3 + EP1_sp3)*2) - (_cons + sp1*2 + sp2*2 +sp3*2)

**Adding covariates in adjusted models**

It is assumed that covariates are binary in nature (i.e., 0 or 1) with multiple categories by dummy variable creation.

For example, if adjusting for the variable **home** owner ship (yes = 1, no = 0).

**Code**

🡪 gen home_sp1=home*sp1

🡪 gen home_sp2=home*sp2

🡪 gen home_sp3=home*sp3

**Rerun as adjusted model:**

xtmixed SDQ EP1 sp1 sp2 sp3 EP1_sp1 EP1_sp2 EP1_sp3 home home*sp1 home*sp2 home*sp3 || ID: sp1 sp2 sp3

rerun lincom code above to calculate estimates.
